# Macrophage infiltration in 3D cancer spheroids to recapitulate the TME and unveil interactions within cancer cells and macrophages to modulate chemotherapeutic drug efficacy

**DOI:** 10.1186/s12885-023-11674-9

**Published:** 2023-12-07

**Authors:** Khushwant Singh, Pramod K. Gautam

**Affiliations:** Department of Biochemistry, All India Institute of Medical Sciences, New Delhi, 110029 India

**Keywords:** Tumor microenvironment, Extracellular matrix, Cysteine proteases, Hypoxia, Tumor associated macrophage polarization, Warburg effect, Inflammation, Doxorubicin

## Abstract

**Background:**

Recapitulating the tumor microenvironment (TME) in vitro remains a major hurdle in cancer research. In recent years, there have been significant strides in this area, particularly with the emergence of 3D spheroids as a model system for drug screening and therapeutics development for solid tumors. However, incorporating macrophages into these spheroid cultures poses specific challenges due to the intricate interactions between macrophages and cancer cells.

**Methods:**

To address this issue, in this study, we established a reproducible healthy multicellular 3D spheroid culture with macrophage infiltrates in order to mimic the TME and modulate the drug’s efficacy on cancer cells in the presence of macrophages. A 3D spheroid was established using the human cancer cell line CAL33 and THP1 cell derived M0 macrophages were used as a source of macrophages. Cellular parameters including tumour metabolism, health, and mitochondrial mass were analysed in order to establish ideal conditions. To modulate the interaction of cancer cells with macrophage the ROS, NO, and H_2_O_2_ levels, in addition to M1 and M2 macrophage phenotypic markers, were analyzed. To understand the crosstalk between cancer cells and macrophages for ECM degradation, HSP70, HIF1α and cysteine proteases were examined in spheroids using western blotting and qPCR.

**Results:**

The spheroids with macrophage infiltrates exhibited key features of solid tumors, including cellular heterogeneity, metabolic changes, nutrient gradients, ROS emission, and the interplay between HIF1α and HSP70 for upregulation of ECM degradading enzymes. Our results demonstrate that tumor cells exhibit a metabolic shift in the presence of macrophages. Additionally, we have observed a shift in the polarity of M0 macrophages towards tumor-associated macrophages (TAMs) in response to cancer cells in spheroids. Results also demonstrate the involvement of macrophages in regulating HIF-1α, HSP70, and ECM degradation cysteine proteases enzymes.

**Conclusions:**

This study has significant implications for cancer therapy as it sheds light on the intricate interaction between tumor cells and their surrounding macrophages. Additionally, our 3D spheroid model can aid in drug screening and enhance the predictive accuracy of preclinical studies. The strength of our study lies in the comprehensive characterization of the multicellular 3D spheroid model, which closely mimics the TME.

**Supplementary Information:**

The online version contains supplementary material available at 10.1186/s12885-023-11674-9.

## Background

In recent years, there have been significant strides in the area of cancer research, particularly with the emergence of 3D spheroids as a model system for drug screening and therapeutics development for solid tumour [1]. Spheroids are three-dimensional (3D) cell culture models that mimic the architecture and microenvironment of solid tumours more closely than traditional two-dimensional (2D) monolayer cultures [1]. Tumour spheroids are generated by culturing cancer cells in a 3D environment, allowing them to self-assemble into multicellular aggregates. This 3D structure enables the formation of cell–cell interactions, leading to the development of complex tumour-like structures [2]. Glucose metabolism plays a crucial role in cancer development and progression of cancer. In the tumour microenvironment (TME), including the immune cells such as macrophages, significantly influences metabolic reprogramming in cancer cells [3]. Glucose is primarily metabolised through glycolysis, leading to the production of pyruvate, which is then converted to lactate rather than being fully oxidised in the mitochondria. Whereas cancer cells exhibit altered glucose metabolism, known as the Warburg effect or aerobic glycolysis, characterised by increased glucose uptake and lactate production, even under oxygen-sufficient conditions [3]. This metabolic phenotype allows cancer cells to meet their energy demands and support rapid proliferation. Macrophages are key immune cells present in the tumour microenvironment and can be polarised into distinct phenotypes, including pro-inflammatory M1 and anti-inflammatory M2 macrophages [4]. Macrophages play a dual role in cancer, with M1 macrophages exhibiting anti-tumour properties, while M2 macrophages promote tumour growth and immune evasion [4]. The metabolic phenotype of macrophages also influences their functions and interactions with cancer cells [3]. Macrophages exhibit a metabolic plasticity that allows them to adapt to different microenvironments [5]. Resting macrophages, known as M0 macrophages, primarily rely on oxidative phosphorylation (OXPHOS) for energy production [3, 6]. Upon activation, M1 macrophages shift towards glycolysis to support their pro-inflammatory functions, while M2 macrophages rely more on OXPHOS to support their immunosuppressive and tissue remodelling functions [6]. Cancer cell-derived factors, such as cytokines (e.g., interleukin-6, tumour necrosis factor alpha) and chemokines (e.g., CCL2, CCL5), can polarise macrophages towards the M2 phenotype, which promotes a shift towards OXPHOS and supports tumour growth [7]. which influences the glucose metabolism of macrophages in the TME. Additionally, cancer cells release lactate, which can be taken up by M2 TAMs and utilised as a fuel source through OXPHOS [8]. The metabolic interplay between cancer cells and macrophages has significant implications in cancer progression. M2 TAMs, fuelled by lactate and oxidative metabolism, promote tumour growth, angiogenesis, and immunosuppression [8]. The metabolic reprogramming of macrophages towards M2 TAMs phenotype can also influence the tumour microenvironment by altering nutrient availability and immune responses, facilitating tumour immune evasion and metastasis [3]. TAMs are known to exhibit diverse phenotypes, including pro-tumoral and immunosuppressive characteristics, which can contribute to reduce efficacy of chemotherapeutic drugs which is referred to as TAM-Mediated chemo resistance [9]. Cells in TME secrete various growth factors, cytokines, and chemokines that promote tumour cell survival and proliferation. TAMs upregulate the expression of drug efflux pumps, such as P-glycoprotein, which actively transport chemotherapeutic agents out of cancer cells [10]. This efflux mechanism reduces intracellular drug concentrations and limiting their cytotoxic effects. Furthermore, TAMs re-model the TME by secreting extracellular matrix components and promoting angiogenesis which alters TMEs architecture, create physical barriers and hinder drug penetration into the tumour, reducing drug efficacy [11]. Incorporating macrophages into tumor spheroids to study the interaction between cancer cells and macrophages poses distinct challenges due to the intricate nature of their interactions. Optimising the macrophage-to-cancer cell ratio and culturing conditions is crucial to maintain spheroid integrity and functionality [12]. In this article we have explored the potential strategy to co-culture macrophages in tumour spheroids to recapitulating key features of solid tumours, including cellular heterogeneity, glucose metabolism, nutrient gradients, ROS emission, and interplay between hypoxia and HSP70 for ECM degradation enzyme upregulation to making a more physiologically relevant spheroid model for drug Screening.

## Material method

### Materials

Dulbecco’s MEM (DMEM), Roswell Park Memorial Institute medium (RPMI 1640), fetal bovine serum (FBS), and antibiotic antimitotic were purchased from Gibco BRL (Gaithersburg, MD, USA). Phorbol-12-myristate-13-acetate (PMA), DCFDA (Cat. No. D6883), and titanium(IV) oxysulfate solution (495,379) were purchased from Sigma Aldrich (USA). For western blotting, anti-human HSP70 (Cat. No. MA3-008), HIF1α (Cat. No. H1alpha67), cathapsin B (Cat. No. SC365558), cathapsin L (Cat. No. SC32320), and HRP-Goat anti-Mouse IgG (Cat. No. 62–6820) antibodies were used. For flow cytometry, anti-human CD44 (Cat. No. 338819), CD24 (Cat. No. 329505), CD14 (Cat. No. 130113–705), CD86 (Cat. No. 130–114-099), CD206 (Cat. No. 130–123-920), and CD163 (Cat. No. 130–123-310) antibodies were used. CalcineAM (Cat. No. 13529), Mitospy (Cat. No. 50–207-9911), CFSE (Cat. No. 423801) were purchased from Biolegend (San Diego, USA) and DAPI Solution (Cat. No. 62247) and Cobalt(II) chloride (Cat. No. AC214130050) were purchased from Thermo Fisher Scientific (Massachusetts, US). Nile red dye (Cat. No. 47353) and Griess Reagent (Cat. No. 35657) were purchased from SISCO Research Laboratories (Mumbai, India). Glucose Colorimetric Detection Kit (Cat. No. ab282922) and Lactate Colorimetric Assay Kit (Cat. No. ab282923) were purchased from abcam (Cambridge, UK). Clarity Western ECL Substrate (Cat. No. 1705061) and 2. Nitrocellulose Membrane (Cat. No. 1620112) were purchased from Biorad (California, USA). Ultralow attachment plate Akura™ 96 was obtained from InSphero (Wagistrasse, Switzerland), and other plastic wares were purchased from Corning (New York, USA). All other chemicals used in this study were of Analytical AR/Excel AR grade and were purchased from Thermo Fisher Scientific (Massachusetts, US), Merck Laboratories (Rahway, New Jersey, USA), SISCO Research Laboratories (Mumbai, India), and HiMedia Laboratories Private (Mumbai, India).

## Methods

### Cell lines culture

CAL33 cancer cells were obtained from ATCC (Virginia, USA) and THP1 cells were obtained from the NCCS (Pune, India). The CAL33 cells were maintained in high glucose DMEM medium and THP1 cells were maintained in RPMI1640 medium. Both media supplemented with 10% FBS and 1% penicillin/streptomycin (Thermo Fisher Scientific Inc., MA, USA). The cells were incubated at 37 °C in a humidified incubator with 5% CO2.

### Macrophage polarization

To induce polarization of THP1 monocytes to macrophages, phorbol-12-myristate-13-acetate (PMA) was used. THP1 monocytes were cultured in RPMI-1640 medium supplemented with 10% FBS and 1% penicillin/streptomycin. The cell clumps were dissociated, and single cell suspension thus obtained was centrifuged. The cells pallet was dissolved and cultured for 48 h in a complete RPMI1640 medium with PMA at a concentration of 150 ng/ml.

### Spheroid culture

The CAL33 cancer cells were washed with 1X PBS and incubated with 0.05% trypsin followed by neutralization of trypsin with FBS containing media and centrifuge at 1500 rpm for 5 min. The cell pallet was dissolved in FBS containing DMEM media and cell viability was determined using trypan blue dye. To establish a spheroid of CAL33 cells, only 7500 viable CAL33 cells were seeded in ultralow attachment plate and incubated at 37 °C in 5% CO2 incubator. The macrophages in the TME can range upon 50% of whole tumor mass. To establish a heterogeneous spheroid of CAL33 with macrophages, a ratio of 3:1 (CAL33 cells and M0 macrophages) was taken. In brief 2500 viable M0 macrophages were mixed with 7500 of viable CAL33 cells and seeded in ultralow attachment plate followed by incubation at 37 °C in 5% CO2 incubator.

### Glucose consumption

The glucose consumption assay was used to determine the rate of glucose uptake and utilisation by cells in spheroids with and without macrophages. After 24 h, spheroids were washed with 1XPBS twice to remove any residual glucose and culture media was replaced by 100 µl of glucose-free media for 2 h to create glucose starvation. Following 2 h of incubation the volume of culture media was increased to 200 µl by adding 100 µl of 9 mg/ml (50 mM) glucose media and incubated at 37 °C in a CO2 incubator. The spheroids and conditioned media were harvested at different days to determine the glucose consumption at different days. For determining the glucose in conditioned media, Glucose Colorimetric Detection Kit was used and glucose standard curve of 0–4.5 mg/ml mM was prepared by serially diluting 4.5 mg/ml glucose in glucose-free cell culture media. Glucose concentration in each conditioned media was determined within 30 min of harvest and interpolated from a standard curve drawn using the absorbance values of the standard solutions and their respective concentrations in GraphPad prism.

### Lactic acid release assay

The same conditioned media harvested for glucose consumption assay was used for lactic acid release assay. For determining the lactic acid in conditioned media, Lactate Colorimetric Assay Kit was used. Sodium lactate standard curve of 0–2 μg/ml or 18 mM was prepared by serially diluting 2 μg/ml sodium lactate in glucose and lactic acid-free media. Lactic acid concentration in each conditioned media was determined within 30 min of harvest and interpolated from a standard curve drawn using serially Lactic acid standard solutions in GraphPad prism.

### Cellular health

CalcineAM is a non-fluorescent dye that can penetrate cell membranes. Once inside live cells, it undergoes hydrolysis by intracellular esterases to produce green-fluorescent Calcein. The cultured spheroids with and without macrophages were washed with 1XPBS and stained with 1 mM CalcineAM solution diluted in serum-free cell culture medium with 1 µg/ml DAPI and incubated in 37 °C incubator for 30 min. The CalcineAM solution was removed from spheroids and spheroids were incubated in 10% serum media for 30 min followed by imaging with confocal microscope. The spheroids were dissociated with 0.05% trypsin and stained with 1 mM CalcineAM solution diluted in serum-free cell culture medium and CalcineAM fluorescence intensity were determined at 491 nm excitation and 516 nm emission using fluorescence microplate reader. The mean florescence intensity normalised with blank media plotted in GraphPad prism.

### Mitochondrial mass assay

This Mitospy is cell permeable fluorescent probe that selectively stains mitochondria of live cells and used for quantification of mitochondrial content of live cells. To determine the mitochondrial mass of cells, the spheroids were dissociated and stained with 1 mM Mitospy solution in serum-free cell culture medium and incubated in 37 °C incubator for 30 min. Following the incubation, the cells were washed with 10% serum media and Mitospy green fluorescence intensity was determined at 491 nm excitation and 516 nm emission using fluorescence microplate reader. The mean florescence intensity normalised with blank media plotted in GraphPad prism.

### Cell viability

DAPI (4', 6-diamidino-2-phenylindole) is a fluorescent DNA-binding dye that only permeates dead cells. It exhibits stronger fluorescence when bound to DNA in dead cells with intact nuclear membranes. To determine the cell viability of cells, the spheroids were dissociated and stained with 1 µg/ml DAPI solution in PBS and incubated in 37 °C incubator for 5 min. Following the incubation, the cells were washed with 10% serum media and DAPI-blue fluorescence intensity was determined at 359 nm excitation and 457 nm emission using fluorescence microplate reader. The mean florescence intensity normalised with blank media plotted in GraphPad prism.

### Cytotoxicity assay

The cell proliferation in this study was measured using the MTS assay using CellTiter 96 Aqueous One Solution Cell Proliferation kit. The spheroids on 5^th^ day were treated with doxorubicin for 24 h followed by dissociation of spheroid with 0.05% trypsin. Total 10^4^ cells were seeded in each well of a 96-well plate and incubated in a CO2 incubator and MTS solution in each well was added as per kit instruction manual. Percent cell viability in each well was calculated using the following equation:

$$Percent\, cell \,viabilty =\frac{Treated \,well \,OD \,- \,Blank \,control \,OD}{Untreated \,control \,OD \,- \,Blank \,control \,OD}\times 100$$

GraphPad prism software was utilized to plot the percent cell viability versus drug concentration curve and to calculate inhibitory concentration 50 (IC_50_).

### DCFDA ROS

The DCFDA (2',7'-dichlorodihydrofluorescein diacetate) is a non-fluorescent compound that can freely penetrates the cell membrane. Once inside the cells, it is deacetylated by cellular esterases and oxidised by ROS to form the highly fluorescent compound DCF (2′,7′-dichlorofluorescein). To determine the ROS level in cells, the spheroids were dissociated and stained with 1 mM DCFDA solution in PBS and incubated in 37 °C incubator for 20 min. Following the incubation, the cells were washed twice with 1XPBS and DCFDA fluorescence intensity was determined at 491 nm excitation and 516 nm emission using fluorescence microplate reader. The mean florescence intensity normalised with blank media plotted in GraphPad prism.

### Grease test

The Griess test was used for the estimation of nitric oxide (NO) levels in conditioned media samples. The conditioned media collected from the spheroids were transferred to a clean microplate and incubated with equal volume of Griess Reagent on shaker at room temperature for 10 min to allow the Griess reaction to occur. To determine the NO level, the absorbance at 540 nm was recorded. To interpolate concentrations of NO, serial dilution of nitrite was prepared in serum free media and processed along with the conditioned media samples. A standard curve was drawn using the absorbance values of the standard solutions and their respective concentrations in GraphPad prism.

### Hydrogen peroxide (H_2_O_2_) release

The TiOSO_4_ or titanium sulphate assay, was used for the estimation of hydrogen peroxide (H_2_O_2_) concentration in the conditioned media samples. The reaction between H_2_O_2_ and titanium sulphate leads to form a yellow-coloured complex which represents the concentration of H_2_O_2_ in the sample. The conditioned media collected from the spheroids were transferred to a clean microplate and incubated with equal volume of titanium sulphate solution on shaker at room temperature for 10 min to allow the colour development. Following the colour development, the reaction was terminated to stabilise the yellow complex by adding equal volume of 2% sulphuric acid solution to each cuvette or test tube to stop the reaction. To determine the H_2_O_2_ release, the absorbance at 410 nm was recorded. To interpolate concentrations of H_2_O_2_ serial dilution (0–0.1 M) of H_2_O_2_ was prepared in serum free media and processed along with the unknown samples. A standard curve was drawn using the absorbance values of the standard solutions and their respective concentrations in GraphPad prism.

### CFSE cell staining

Carboxyfluorescein Succinimidyl Ester (CFSE) is a cell-permeable fluorescent dye that covalently binds to intracellular proteins, resulting in the labelling of all cellular cytoplasmic components. To label cancer cells for confocal microscopy, CAL33 cells were stained with 1 µM CFSE solution in serum-free cell culture medium and incubated in 37 °C incubator for 15 min. Following the incubation, the cells were washed twice with 5% serum media and processed for spheroid culture.

### Nile red staining

To locate monocytes/macrophages in spheroids, monocytes were labelled with Nile red for confocal microscopy. Nile red is a fluorescent dye that binds to the intracellular lipid droplets and lipid bilayer of live cells. The M0 macrophages were stained with 1 µg/ml Nile red solution in serum-free cell culture medium and incubated in 37 °C incubator for 15 min. Following the incubation, the cells were washed twice with 5% serum media and processed for spheroid culture.

### Immunocytochemistry microscopy

The cells were cultured on sterile round coverslips and then fixed in 4% formalin for 10 min. To reduce non-specific binding of antibodies, the cells were incubated in a 1% BSA solution for 20 min. The coverslips with the cells were then incubated overnight at 4ºC with primary antibody. The next day, cells were washed and permeabilized with 0.1% Triton X-100 for 5 min, and the cell nuclei were stained with DAPI (1 µg/ml) for 5 min at room temperature. Each step was followed by three washes of 5 min each with 1XPBS. Finally, the coverslips were mounted in a glycerol-based mounting media with DABCO as a quenching agent for visualisation under a microscope (ECLIPSE 80I, Nikon Instruments Inc., NY, USA). The image analysis was done using ImageJ software.

### Flow cytometry

The spheroids were washed with 1XPBS twice and dissociated with 0.05% trypsin for 5 min. The trypsin was neutralised by addition of serum supplement media and centrifuged for 5 min at 1500 rpm. The cells pallet was washed with 1XPBS to remove debris and excess media. The cells pallet thus obtained was used for staining in 100 μl PBS with 1% BSA. To identify cancer cells and macrophage cell populations, fluorescently labelled antibodies were added to the cell suspension and incubated for 1 h at room temperature in dark. Afterward, cells were washed with 1XPBS to remove unbound antibodies followed by cells resuspension in PBS buffer for flow cytometry analysis. Flow cytometry was performed on BD LSR Fortessa and the analysis was done using FlowJo software.

### Western blotting

The pellet of 10–20 spheroids were collected, washed and suspended in NP40 lysis buffer (50 mM Tris–HCl pH 7.6, 150 mM NaCl, 5 mM EDTA, 1% NP-40, 0.1% sodium dodecyl sulphate) containing 1X protease inhibitor cocktails and kept on ice for 30 min. After that, the cells underwent a freeze–thaw cycle (freeze at -70 °C and thaw at 37 °C). The resulting cell lysate was collected by centrifugation at 13,000 rpm for 15 min at 4 °C. The supernatant was collected, and the total protein was estimated using the BCA method. A total of 30-50 μg of protein was mixed with 5 × SDS loading buffer and denatured by boiling at 100 °C for 5 min. Subsequently, SDS-PAGE was performed using a stacking gel (4%) and separation gel (10%). The proteins separated by SDS-PAGE were transferred to a nitrocellulose membrane. For western blotting, the membranes were incubated with the primary antibody of HSP70 (1:1000), HIF1α (1:500), Cathepsin B (1:500) and Cathepsin L (1:500) overnight at 4 °C, followed by 2 h of incubation with an HRP-conjugated secondary antibody (1:10,000) at room temperature. Each step was followed by three washes of 10 min each with TBS-t (0.1%). Blots were incubated in ECL for three minutes, and then they were exposed to a maximum of two minutes in the BioRad Gel Doc system to detect chemiluminescence.

### Quantitative real-time PCR

RNAiso Plus (Takara Bio USA) was used to isolate total RNA from cells. cDNA was synthesized from one microgram of RNA using the iScript™ cDNA Synthesis Kit. For real-time quantitative PCR the primers were purchased from IDT (Coralville, US). The reactions for Homo sapiens HIF1α (Forward 5′—GGTACTTTACGCACCCTGCT—3′ and Reverse 5′—GCTAGGGCAGGAAAGAGCAA—3′), HSP70 (Forward 5′ -AGGACATCAGCCAGAACAAG- 3′ and Reverse 5′ -CTGGTGATGGACGTAGAAG- 3′), Cathepsin B (Forward 5′ -TGTAATGGTGGCTATCCTGCT- 3′ and Reverse 5′ -AGGCTCACAGATCTTGCTACA- 3′), and Cathepsin L (Forward 5′ -GACTGAGGAATCCTATCCA- 3′ and Reverse 5′ -AAGGACTCATGACCTGCATCAA- 3′) were performed on the Agilent AriaMx Real-time PCR System (California, US) as per recommended protocol using the Brilliant II SYBR® Green qPCR Master Mix (Agilent). GAPDH (Forward 5′—ATGGGGAAGGTGAAGGTCGG—3′ and Reverse 5′—ATGACCTTGCCCACAGCCTT—3′) was used as an internal reference and each sample were set up in triplicate.

## Results

### PMA mediated polarization of THP1 monocytes to M0 macrophages and spheroid establishment

**Fig. 1:**
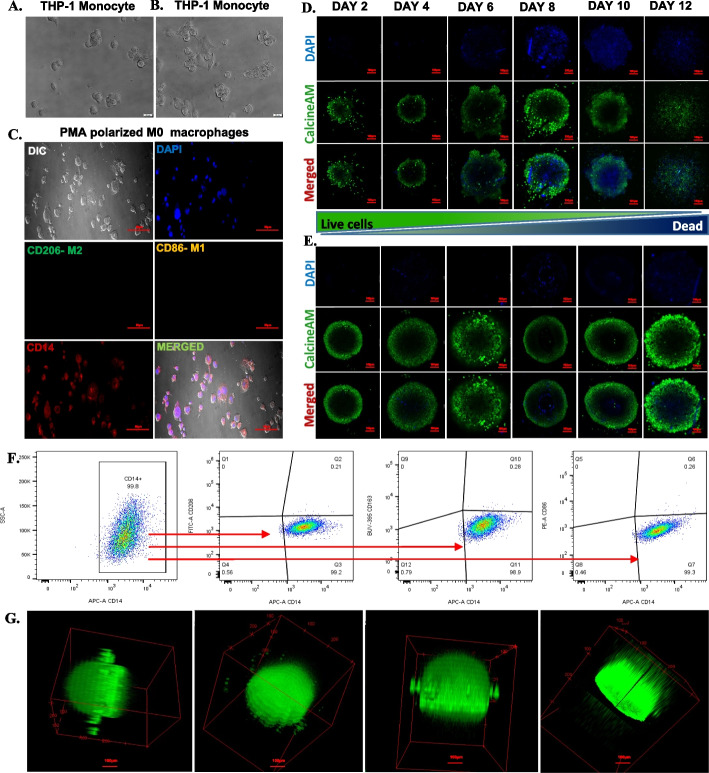
**A** Undifferentiated THP-1 monocytes before treatment with PMA, **B** THP-1 cells at 48 h incubation with 150 ng/ml of PMA to differentiate monocytes to M0 macrophages, **C** M0 macrophage at 48 h fixed and immunolabelled with M1 macrophage marker CD163 (Green), M2 macrophage marker CD207 (yellow), Macrophage marker CD14 (Red), and nuclei stained with DAPI (blue), **D** CalcineAM (Green) and DAPI (Blue) stained CAL33 spheroids without macrophages on different days (at 10X magnification), **E** CalcineAM (Green) and DAPI (Blue) stained CAL33 spheroids with macrophages on different days (at 10X magnification), **F** Flow cytometry analysis for M0 macrophage cell surface marker CD14 with M1 macrophage markers (CD14, CD86) and M2 macrophage markers (CD14, CD206 and CD163) on day 2, **G**. Z- scan confocal microscopy (at 10X magnification) of Spheroids for CalcineAM penetration

The differentiation of THP1 monocytes into M0 macrophages was determined by morphological characterisation, immunocytochemistry (ICC) and flow cytometry. Representative images of THP1 cells and adherent M0 macrophages are presented in Fig. 1 A, B. Undifferentiated THP-1 human monocytes displayed small clusters with round spherical morphology (Fig. 1 A). Post 48 h of PMA treatment, the monocytes got adhered and exhibited amoeboid morphology with cytoplasmic extensions (Fig. 1B). ICC used to determine macrophage surface markers demonstrated that the adhered macrophages were positive for monocyte lineage marker CD 14, while being negative for the M1 (CD 86) and M2 (CD 206) macrophage surface markers (Fig. 1 C). The expression of CD14 indicating their monocyte origin. Further the flow cytometry analysis revealed M0 polarity of adhered macrophages, as indicated by their high expression of cell surface marker CD14 and lack of M1 macrophage marker CD86, as well as M2 macrophage markers CD163 and CD206 expression (Fig. 1 F). The tumour spheroid culture with macrophage was established using 3:1 (7500:2500) ratio of CAL33 cells and M0 macrophages as mentioned in the methodology section. The spheroid with and without macrophages were stained with CalcineAM and DAPI on day 2 to day 12 followed by confocal microscopy imaging. The confocal microscopy image analysis revealed irregular round morphology of spheroids without macrophages, while those with macrophages demonstrated a round spherical morphology as shown in Fig. 1 (D-E). The Z-scan confocal microscopy of CalcineAM stained spheroids confirms the CalcineAM penetration throughout the spheroid as shown in Fig. 1G and Supplementary Fig. 1. Furthermore, CalcineAM staining results revealed that as spheroids without macrophages grow in size, the cellular esterase activity decreases significantly and the number of dead cells increases. On the other hand, the spheroids with macrophages did not depict any significant decrease in esterase activity and the number of dead cells were also significantly lower in comparison to the spheroids without macrophages.

### Macrophage mediated nutritional partition of glucose in 3D spheroids improving overall survival of cancer cells

**Fig. 2:**
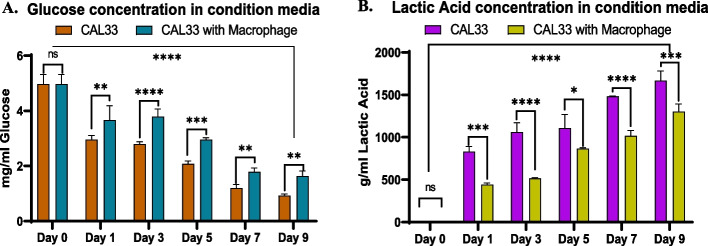
**A** Graph representing left over glucose in conditioned media collected from spheroids with and without macrophages on day 0 to day 9, **B** Graph representing lactic acid secretion by spheroids with and without macrophages in conditioned media collected from day 0 to day 9. The measurements were performed for all the conditions in triplicates (*n* = 3, with conditioned media collected from 3 spheroids in each condition). Statistical significance was analyzed using two-way ANOVA with Sidak's multiple comparisons test and the p-value obtained are indicated (ns *p* =  > 0.9999, ** *p* = 0.0011, *** *p* = 0.0001, **** *p* =  < 0.0001)

In the tumour microenvironment (TME), cancer cells are known to consume more glucose and metabolise it through anaerobic glycolysis, even in the presence of sufficient oxygen [13]. However, glucose is also an important metabolite for non-tumorigenic cells like immune cells in the tumour microenvironment [3, 14]. Despite regular change of cell culture media every 48 h, increase in cell death in spheroid without macrophage was observed. The increase in cell death in spheroids can be due to the lack of nutrients and increased hypoxia [15]. Further to determine the glucose consumption and lactic acid release by the spheroids, the presence of glucose and lactic acid release was determined in the spheroid conditioned media from day 0 to day 9. The spheroids were grown in mixture (1:1) of DMEM and RPMI160 culture media supplemented with glucose at concentration of 5 mg/ml with no lactic acid. The spheroid conditioned media collected at day 1 from spheroid without macrophages demonstrated presence of 3.2 mg/ml of glucose and 877 µg of lactic acid (Fig. 2 A). Following day 1 to day 9 significant reduction in glucose concentration with increase in lactic acid release was observed in spheroid without macrophages (Fig. 2 A, B). Whereas in spheroid with macrophage, similar trend of reduction in glucose concentration with increase in lactic acid was observed from day 1 to day 9. Despite having same number of cancer cells in both spheroids, the amount of glucose consumed, and lactic acid released by spheroid with macrophage was significantly lower as compared to cancer cell spheroids (Fig. 2 B). This analysis of the spheroid conditioned media collected from spheroids with and without macrophages at different time intervals demonstrated that spheroids without macrophages consumed more glucose compared to those with macrophages. Similarly, the increased glucose consumption leads to the excessive production of lactic acid by anaerobic glycolysis as per Warburg hypothesis [13]. The production of lactic acid via anaerobic glycolysis results in an acidic tumor microenvironment, which may increase cell death [8]. One possible reason for increased cell death in spheroids without macrophages could be the rapid proliferation rate of cancer cells in the acidic microenvironment at the core of the spheroid, coupled with the lack of nutrient and gas exchange due to the compactness of the spheroid.

### Infiltrated macrophages in spheroid-improving cancer cell esterase activity and enhancing mitochondrial mass

**Fig. 3:**
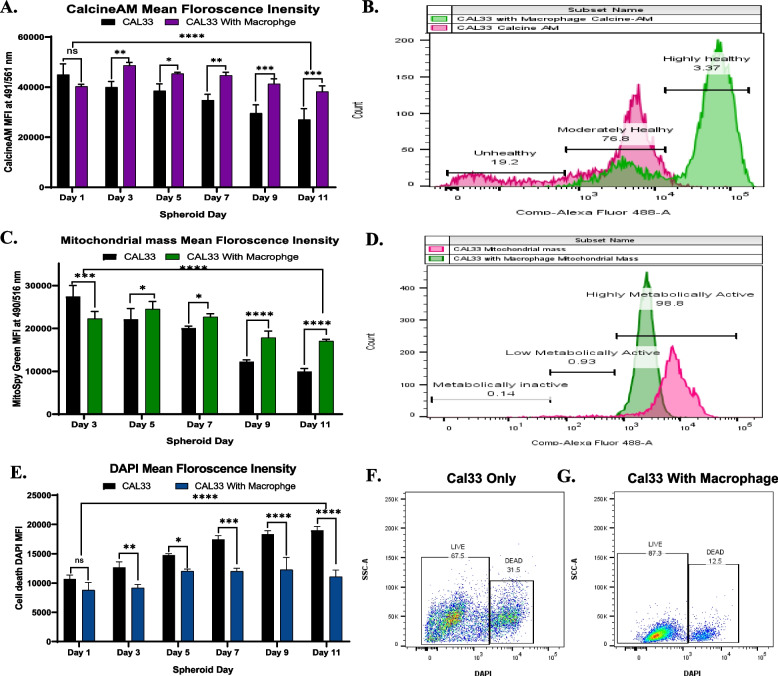
**A** Graph representing CalcineAM mean florescence intensity in single cell suspension of CAL33 spheroids with and without macrophages from day 1 to day 11, **B** CalcineAM flow cytometry analysis layover histogram of spheroids single cell suspension at day 7 with 3 different population of cells representing cellular health status, **C** Graph representing Mitospy-green mean florescence intensity for Mitochondrial mass analysis in single cell suspension of spheroids on day 3 to day 11, **D** Mitospy-green flow cytometry analysis layover histogram of spheroids single cell suspension at day 7 with 3 different population of cells representing different mitochondrial mass and metabolic activity, **E** Graph representing DAPI mean florescence intensity for dead cell analysis in single cell suspension of spheroids with and without macrophages from day 1 to day 11, **F**,** G** Flow cytometry analysis for live cell and dead cell in spheroids single cell suspension at day 7. The measurements were performed for all the conditions in triplicates (*n* = 3, with 4 spheroids single cell suspension in each condition). Statistical significance was analyzed using two-way ANOVA with Sidak's multiple comparisons test and the p-value obtained are indicated (ns *p* = 0.1619, * *p* = 0.0223, ** *p* = 0.0014, *** *p* = 0.0003, *** *p* = 0.0005)

Cancer cells have rapid proliferation rate and require a high amount of energy, leading to increased consumption of glucose and other nutrients [16, 17]. The glucose consumption and lactic acid release assay revealed less glucose consumption in spheroids with macrophages. Further to investigate the impact of macrophage presence on cancer cell in the spheroids, the cellular health was analysed from day 1 to day 11. The cellular health of cells in spheroids was analysed with CalcineAM dye to assess cellular esterase activity. Due to the difference in the cell number in spheroids from day 1 to day 11, the spheroids were dissociated and 5000 live cells in each condition were stained with CalcineAM green dye as mentioned in the methodology section. The spheroids without macrophages demonstrated a significant decrease in esterase activity from day 5 to day 11 of culture, indicating a degradation in cellular health as the spheroid size increased (Fig. 3 A, B). However, spheroids with macrophages did not demonstrate any significant change in esterase activity after 11 days of culture, suggesting that the introduction of macrophages in spheroids helps in maintaining the health of cancer cells (Fig. 3 A, B). In cancer, as the tumour size increases, hypoxia (lack of oxygen) also increases in the core of the tumour. Cancer cells adapt to hypoxia by remaining healthy but becoming less metabolically active. Mitochondria within the cells serve as the energy source for generating ATP. The mitochondrial mass of cells in spheroids was analysed with MitoSpy green dye from day 3 to day 11 to determine metabolic activity as mentioned in the methodology section. The analysis of mitochondrial mass demonstrated that as spheroids without macrophage grow from day 2 to day 11, the mitochondrial mass reduce drastically, making the cancer cells less metabolically active (Fig. 3 C, D). In contrast, spheroids with macrophages did not exhibit any significant decrease in mitochondrial mass in the first 7 days of culture, but a decrease in mitochondrial mass was observed post 8 days of culture (Fig. 3 C, D). However, compared to spheroids without macrophages, spheroids with macrophages displayed a significantly higher amount of mitochondrial mass until day 11, indicating that the introduction of macrophages keep the cancer cells metabolically active. Additionally, cell viability in the spheroids was also assessed using DAPI as mentioned in the methodology section. The viability assay revealed that as the spheroids without macrophages increased in size from day 1 to day 11, cell death also increased, with the observed highest cell death on day 11 (Fig. 3 E). In contrast, spheroids with macrophages showed a slight increase in cell death in the first 5 days of culture, but no significant change in cell viability was observed between day 5 and day 11 of culture. This suggests that macrophage infiltration not only improves cancer cell health and metabolic activity but also contributes to the survival and progression of cancer cells in the tumour microenvironment by limiting cell death. The flow cytometry performed on day 7 as demonstrated in Fig. 3 F, G further support these findings.

### Infiltrated macrophages in spheroid-reducing inflammation

**Fig. 4:**
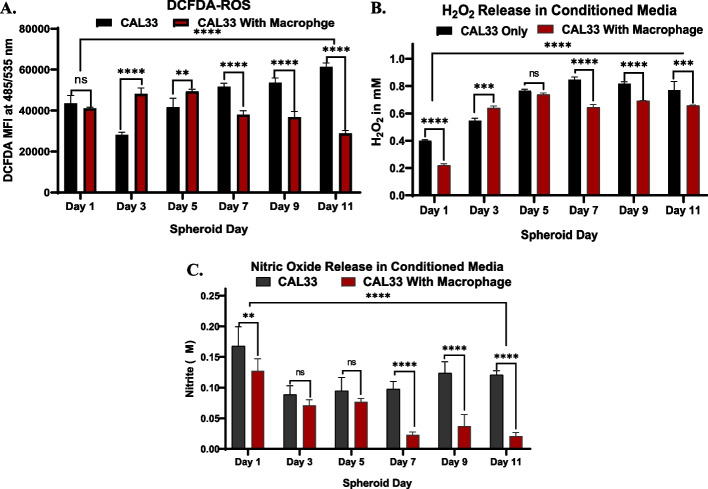
**A** Graph representing DCFHDA-green mediated reactive oxygen species (ROS) mean florescence intensity in single cell suspension of CAL33 spheroids with and without macrophages from day 1 to day 11, **B** Graph representing titanium(IV) sulfate (TiSO4) mediated hydrogen peroxide (H2O2) release in conditioned media collected from CAL33 spheroids with and without macrophages from day 1 to day 11, **C** Graph representing Griess/nitrite test results for nitric oxide (NO) release in conditioned media collected from CAL33 spheroids with and without macrophages from day 1 to day 11. The measurements were performed for all the conditions in triplicates (*n* = 3, with 3 spheroids single cell suspension in each condition). Statistical significance was analyzed using two-way ANOVA with Sidak's multiple comparisons test and the p-values obtained are indicated (ns p = 0.6271, **** *p* =  < 0.0001, ** *p* = 0.0028, **** *p* =  < 0.0001)

In spheroid without macrophage, gradual increase in cell death was observed. In contrast, the spheroids with macrophages demonstrated a slight increase in cell death in the first 5 days of culture followed by no significant change in cell viability between day 5 and day 11 of culture. In the tumour microenvironment (TME), inflammation plays a crucial role in cancer cell survival and immune cell recruitment [18]. When cancer cells die, reactive oxygen species (ROS) are released, leading to oxidative stress [19]. This stress can result in cell death or senescence in neighbouring cancer cells. To investigate the role of ROS in inducing cell death, the level of ROS was assessed using DCFDA. The results revealed that as the spheroid size increased, the level of ROS significantly increased from day 3 to day 11 in spheroids without macrophages (Fig. 4 A). However, in spheroids with macrophages, the trend was reversed; there was an initial increase in ROS levels in the first 5 days of culture, followed by a decrease until day 11 (Fig. 4 A). DCFDA measures the total production of ROS within the cell, while H_2_O_2_ is known to contribute to cell death through processes such as apoptosis, autophagy, necrosis, and ferroptosis. Analysis of the H_2_O_2_ levels in the conditioned media collected from spheroids without macrophages demonstrated a significant increase from day 3 to day 11 of culture (Fig. 4 B). In contrast, in spheroids with macrophages, an initial increase in H_2_O_2_ was observed in the first 5 days of culture, but no significant change in H_2_O_2_ release was observed until day 11 (Fig. 4 B). Apart from ROS and H_2_O_2_, Reactive Nitrogen Species (RNS), especially Nitric Oxide (NO), play a significant role in inducing cancer cell death through apoptosis or necrosis. Quantification of NO in the conditioned media collected on different days indicated a decrease in NO release in the initial 3 days from spheroids without macrophages, followed by an increase until day 11 (Fig. 4 C). However, in spheroids with macrophages, no significant change in NO release was observed in the first 5 days, followed by a significant decrease in NO from day 5 to day 11 (Fig. 4 C).

### Cancer cell polarizing M0 macrophages to the distinct M1 and M2 macrophages phenotype

**Fig. 5:**
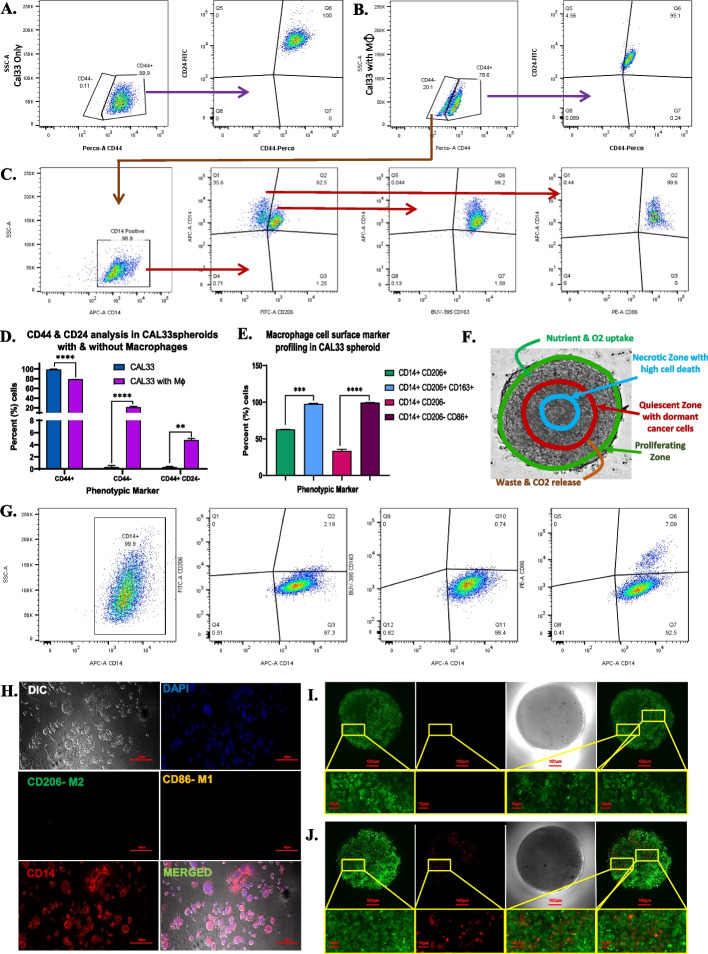
**A** Flow cytometry analysis for cancer cell markers CD44 and CD24 in CAL33 spheroids without macrophages in single cell suspension on day 7, **B** Flow cytometry analysis for cancer cell markers CD44 and CD24 in CAL33 spheroids with macrophages in single cell suspension on day 7, **C** Flow cytometry analysis of CD44 negative cells for M1 macrophage markers (CD14, CD86) and M2 macrophage markers (CD14, CD206 and CD163) in CAL33 spheroids with macrophages in single cell suspension on day 7, **D** Graphical representation of Flow cytometry data for CD44 and CD24 in CAL33 spheroids with and without macrophages, **E** Graphical representation of Flow cytometry data for macrophages cell surface markers for M1 and M2 TAMs in CAL33 spheroids with macrophages, **F** A multicellular spheroid of CAl33 with macrophages representing different zones of TME, **G** Flow cytometry analysis for M0 macrophage cell surface marker CD14 with M1 macrophage marker (CD14, CD86) and M2 macrophage markers (CD14, CD206 and CD163) on day 7, **H** Confocal microscopy images of M0 macrophage on day 7 immuno-labelled with M1 macrophage marker CD163 (Green), M2 macrophage marker CD207 (yellow), Macrophage marker CD14 (Red), and nuclei stained with DAPI (blue), **I** Confocal microscopy image of day 7 CAL33 spheroid without macrophages stained with CFSE-green, **J** Confocal microscopy image of day 7 CAL33 spheroids with macrophages stained with CFSE-green (cancer cells) and Nile red (macrophage)

In early stage of cancer, the number of pro-inflammatory M1 macrophages is higher than anti-inflammatory M2 macrophages [20]. Being pro-inflammatory cells, the M1 macrophages contributes to the cancer cell death by direct cytotoxicity or activation of other immune cells such as T-cells in the tumor microenvironment [21]. The reduction in inflammation in the cancer microenvironment can be due to the presence of anti-inflammatory immune cells or the transformation of resting M0 or inflammatory M1 macrophages into anti-inflammatory M2 macrophages [5]. The reactive oxygen species (ROS), hydrogen peroxide and NO release analysis revealed reduction in the release of these inflammatory molecules following 5 days in spheroid with macrophages. This promoted us to investigate the phenotype of macrophages phenotypes (for M1 and M2) as well as the cancer cells (for cancer stem cells) within the spheroids. Flow cytometry analysis for cancer cells and macrophage cell surface markers were performed on 7-day-old spheroids. To differentiate the cancer cells from macrophages, the cancer cell specific CD44 marker was used whereas CD14 marker was used to identify the macrophage. The results demonstrated that all the cells in the spheroids without macrophages were positive for CD 44 (Fig. 5A). Among these CD44 positive cells, no cell exhibited phenotype similar to cancer stem cells (CSCs) with CD44^high^CD24^low^ markers, whereas 99.9% cells displayed CD44^high^CD24^high^ phenotype or a non-CSCs like phenotype (Fig. 5A). In spheroids with macrophages, two distinct populations were observed—one positive for CD44 and other negative for CD 44 (Fig. 5 B). The observed percentage of CD 44 positive cells was approximately 78% and the percentage of CD 44 negative cells was 22% (Fig. 5B, D). Among the 78% CD44 positive cells, the ratio of CSC-like cells with CD44^high^CD24^low^ markers was 4.5% and remaining 95.1% cells demonstrated CD44^high^CD24^high^ phenotype or a non-CSCs like phenotype (Fig. 5B, D). Within the CD44 negative cells, total 10,000 events were recorded and the analysis of CD 44 negative cells for CD14marker revealed that 98% of these cells were positive for CD14, a macrophage and monocyte marker (Fig. 5C, E). Within this CD14 positive population, approximately 62.5% cells were positive for M2 macrophage marker CD 206 while 35.6% were negative for CD 206 (Fig. 5C, E). Among the 62.5% of CD14 and CD206 dual positive cells, approximately 98% cells were also expressing CD163, a phenotypic marker of M2 TAM macrophages as shown in Fig. 5C, E. In the remaining 35.6% of CD14 positive and CD206 negative cells, 99% cells were positive for CD86, a phenotypic marker of M1 macrophages (Fig. 5C, E). In contrast, the analysis of Thp1-derived M0 macrophages did not demonstrate expression of CD 206, CD163 at 7^th^ days of culture, but 7% of cells demonstrated CD86 positive phenotype at 7^th^ days of culture (Fig. 5G, H). Further to localize macrophages within the spheroids, CAL33 cells were stained with CFSE-green dye and macrophages were stained with Nile red dye and seeded in ultralow attachment plate followed by culture for 7 days. Live cell confocal microscopy imaging of spheroids with and without macrophages on day 7 revealed the presence of macrophages at various locations within the spheroid (Fig. 5 I), while the CFSE-green dye stained CAL33 cells spheroid without macrophages (Fig. 5 J) demonstrated round morphology with no macrophages. The ICC and flow cytometry results together confirm the co-existence of both M1 and M2 macrophages in the spheroid and tumour cell mediated polarisation of M0 macrophages towards M1 and M2 phenotypes for cancer progression. Figure 5F depicts a representative image of CAL33 spheroid with macrophage infiltration, illustrating distinct zones of proliferation, quiescent and hypoxic core within the cancer spheroid.

### Macrophage infiltrated spheroids enhancing ECM degrading proteases and HSP70 for stabilization of HIF1α

**Fig. 6:**
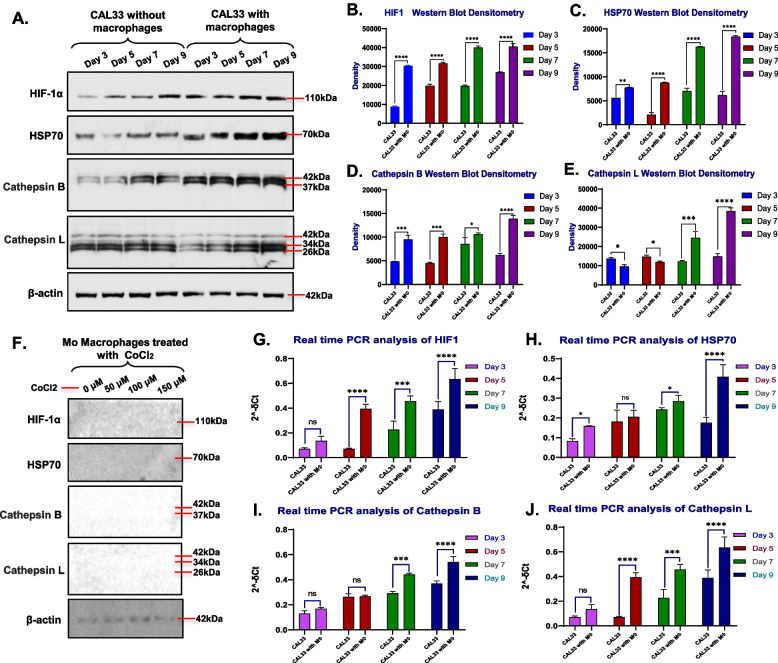
**A** Western blot analysis for HIF-1 α, HSP70, Cathepsin B and Cathepsin L with β-actin in protein lysate collected from CAl33 spheroids with and without macrophages on day 3, 5, 7 and 9, **B-E** Densitometry analysis for HIF-1 α, HSP70, Cathepsin B and Cathepsin L in western blots normalized with β-actin as a positive control (*n* = 2), **F** Western blot analysis for HIF-1 α, HSP70, Cathepsin B and Cathepsin L with β-actin in protein lysate collected from 25,000 M0 macrophages treated with CoCl2 at 0 μM, 50 μM, 100 μM, and 150 μM for 48 h to induce hypoxia to mimic condition like spheroid with macrophage. **G-J** Real time PCR analysis for HIF-1 α, HSP70, Cathepsin B and Cathepsin L in mRNA isolated from CAl33 spheroids with and without macrophages on day 3, 5, 7 and 9. The western blots were performed for all the conditions in duplicate (*n* = 2, with 10 spheroids in each condition). Statistical significance was analyzed using two-way ANOVA with Sidak's multiple comparisons test and the p-value obtained are indicated (ns *p* = 0.5, * *p* ≤ 0.01, ** *p* ≤ 0.001, *** *p* ≤ 0.0001, **** *p* ≤ 0.00001

As the above results suggests that the over growth of cells in tumor leads to increase in size and decreased nutrition supply which alters cancer cell health with lower mitochondrial mass along with the increased inflammation, and cell death in spheroid without macrophages. The opposite results were observed in spheroid with macrophages. Under hypoxic conditions and to overcome the inflammation, the cancer cells increase the release molecules that induce the shift in polarity of M0 or inflammatory M1 macrophages to anti-inflammatory M2 macrophages which reduces inflammation in tumor microenvironment [5]. In addition, the cancer cells also increase the expression of molecular chaperons such as HSP70 [22]. The interaction between HIF-1α and HSP70 stabilises HIF-1α, preventing its degradation and allowing it to accumulate [22]. This accumulation leads to the activation of genes involved in angiogenesis, metastasis, and inhibition of the apoptosis [23]. In addition, increase in CSCs like cells with CD44^high^CD24^low^ markers promote metastasis and cancer cells themselves in TME secrete cysteine proteases that degrade the extracellular matrix, in addition to the proteolytic enzymes secreted by M2 TAMs which further accelerate ECM degradation, tumour cell invasion and migration. In light of above-mentioned results and flow cytometry findings, we decided to assess the expression levels of HIF-1α, HSP70, and cysteine proteases cathepsin B and L in spheroids. The results of western blot and its densitometry normalised with beta actin conducted on day 3, 5, 7, and 9 revealed a significant high expression of HIF-1α in spheroids with macrophages, reaching its peak at day 9 (Fig. 6A, B). In spheroids without macrophages, HIF-1α expression also increased from day 5 to day 9, but the level was significantly lower compared to spheroids with macrophages (Fig. 6A, B). Additionally, no significant change in HSP70 expression was observed in spheroids with macrophages until day 5 (Fig. 6A, C). However, at day 7 and 9, an increase in HSP70 expression was observed (Fig. 6A, C). In contrast, spheroids without macrophages demonstrated an initial increase in HSP70 expression post 5 days of culture, followed by a decrease possibly due to more cell death.

For the cysteine proteases cathepsin B and L, western blot densitometry results normalized with beta-actin demonstrated an increase in the expression of cathepsin B from day 3 to 7, followed by a decrease at 9-day culture in spheroids without macrophages (Fig. 6A, D). Conversely, spheroids with macrophages also exhibited an increase in cathepsin B expression, but the change was notably insignificant from day 3 to 7, whereas the cathepsin B expression was significantly higher at day 9 culture compared to spheroids without macrophages (Fig. 6A, D). As for cathepsin L expression, no significant change was observed in spheroids without macrophages until day 9 (Fig. 6A, E). In contrast, spheroids with macrophages demonstrated a significant increase in cathepsin L expression from day 3 to day 9. The changes in expression of HIF-1α, HSP70, and cysteine proteases cathepsin B and L in spheroids with macrophages could be attributed to the presence of macrophages. The lysate for western blotting was prepared from 10 spheroids with 25,00 macrophages in each spheroid or with 25,000 macrophages in the whole lysate. Cobalt chloride (CoCl_2_) was used for inducing hypoxia in cell culture experiments [24]. To determine the expression of HIF-1α, HSP70, and cysteine proteases cathepsin B and L in macrophages, 25,000 M0 macrophages were seeded in a 12-well plate and treated with 0 µM, 50 µM, 100 µM, and 150 µM cobalt chloride (CoCl_2_) to induce spheroid-like hypoxic conditions [25]. The cell lysate was prepared as mentioned in the methodology section, followed by western blotting. As shown in Fig. 6F, the macrophages did not demonstrate any significant change in the expression of HIF-1α, HSP70, and cathepsin B and L post-treatment with CoCl_2_, whereas noticeable expression of β-actin can be seen.

Further to confirm the western blot results, RNA was isolated from spheroids and qPCR for HIF-1α, HSP70, cathepsin B and cathepsin L was performed as mentioned in the methodology section. Consistent with the results of the western blots, the qPCR analysis demonstrated significant increase in the expression of HIF-1α from day 3 to day 9 in both CAL33 spheroids with and without macrophages, with the highest HIF-1α expression on day 9 as shown in Fig. 6G. Similarly, the gradual increase in HSP70 expression was observed in both spheroids. In spheroids without macrophages, an increase in HSP70 expression was observed until day 7, with the peak of expression at day 7, followed by a decrease in expression on day 9. In contrast, spheroids with macrophages demonstrated a peak in HSP70 expression on day 9, as shown in Fig. 6H. Additionally, the expression analysis of cathepsin B in spheroids without macrophages demonstrated increased expression from day 3 to day 9, as presented in Fig. 6I. In comparison to spheroids without macrophages, no significant change in cathepsin B expression was observed until day 5 in spheroids with macrophages. In contrast, a significant increase in cathepsin B expression from day 7 to day 9 was observed in spheroids with macrophages (Fig. 6I). Surprisingly, low expression of cathepsin L was observed at day 3 in both spheroids with and without macrophages. Contradictory to day 3, significantly higher expression of cathepsin L was observed in spheroids with macrophages at day 5, followed by a further increase until day 9. In spheroids without macrophages, no significant change in cathepsin L was observed on day 5, followed by an increase from day 7 to day 9, but at a comparably lower level than the spheroids with macrophages (Fig. 6J).

### Infiltration of macrophages in spheroids decrease the cytotoxicity of chemotherapeutic drugs

**Fig. 7:**
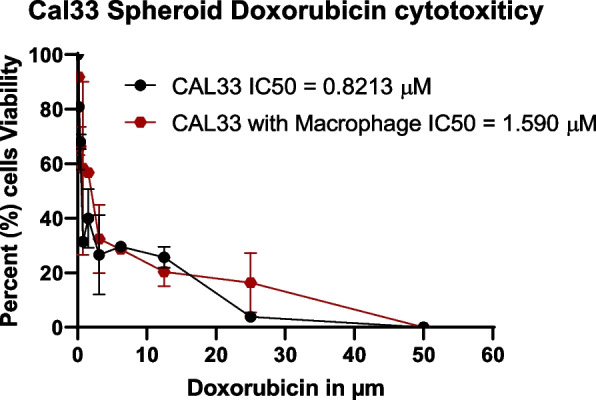
MTS assay result; The effects of Doxorubicin on 5 days old CAL33 spheroids with and without macrophages exposed to 10 ηM to 100 μM/ml serial dilution of Doxorubicin for 24 h The results of the study revealed a gradual decline in the percentage of cell viability as the dose of Doxorubicin increased. To ensure accurate measurements, the absorbance values were adjusted by subtracting the absorbance of DMSO

The results of the MTS assay on CAL33 spheroids with and without macrophages after 24 h are depicted in Fig. 7. It was found that doxorubicin induced complete cell death (100%) in spheroids without macrophages at a dose of 40–50 µM/ml, with an observed IC50 concentration of 0.8213 µM/ml. In spheroids with macrophages, 100% cell death was observed at a dose of 50 µM/ml, with an observed IC50 concentration of 1.590 µM/ml.

## Discussion

Recapitulating the tumor microenvironment (TME) for in vitro studies continues to present significant challenges in cancer research. Over the past two decades, notable advancements have been made, including the emergence of 3D spheroids as a model that can be utilized for drug screening and therapeutics development targeting solid tumours [3]. The primary objective of this study was to establish 3D cancer spheroids containing infiltrated macrophages, which could replicate key characteristics of solid tumours. These features include cellular heterogeneity, metabolism, nutrient gradients, ROS emission, and the interplay between hypoxia and HSP70 for upregulation of ECM degradation enzymes. The findings from the spheroids with macrophage infiltration reveal a round morphology and enhanced esterase activity, along with a reduced number of dead cells compared to spheroids without macrophage infiltration. The results obtained suggest that monocyte-derived M0 macrophages can be employed to create spheroids that resemble the TME. Tumour cells can switch to other nutrient sources in addition to glucose [4]. Some tumour cells are facultative anaerobes and can use oxygen as the primary energy source, while others may rely on fermentation of glycogen, lipids, or amino acids [13, 16, 17]. Furthermore, the analysis of the conditioned media collected from spheroids with and without macrophages demonstrated that spheroids without macrophages drastically consumed more glucose compared to those with macrophages, leading to an acidic tumour microenvironment due to the production of lactic acid. This underscores the importance of the macrophage-tumour interaction in regulating the partitioning of glucose and maintaining an optimal TME. Macrophages have been shown to be important in improving the health and metabolic activity of cancer cells in various ways [3–5, 26]. The results also demonstrated the significant role of macrophage infiltration in regulating the health of cancer cells within spheroids by enhancing esterase activity, metabolic activity, and an increase in mitochondrial mass. These findings highlight the crucial contribution of macrophages in influencing the cellular dynamics and functionality within the spheroid model, shedding light on the potential for therapeutic screening to target tumorigenic cells. Furthermore, the infiltration of macrophages were observed to mitigate cell death within the spheroids, leading to enhanced cell viability. These findings reinforce the notion that macrophage infiltration plays a pivotal role in the progression of cancer cells within the TME [26]. Macrophages, as immune cells, exert significant influence on the tumor microenvironment by secreting various anti-inflammatory cytokines and chemokines that effectively dampen inflammation and facilitate the resolution of the inflammatory response [7, 18, 27]. The inflammation results highlight the crucial role of macrophages in 3D spheroids, as they regulate the production of reactive oxygen species (ROS), hydrogen peroxide (H2O2), and reactive nitrogen species (RNS). These molecules act as signalling molecules that control cancer cell death, senescence, and overall cellular health [19, 28, 29]. Macrophages function as protective agents by counteracting the harmful effects of ROS and promoting spheroid integrity. Furthermore, based on the analysis of DCFDA-ROS, NO, and H2O2, the results indicates that the M0 macrophage within the spheroid polarizes into the M1 macrophage within two to three days of incubation, as suggested by an increase in the analysis of DCFDA-ROS, NO, and H2O2. The polarization of the M1/M0 macrophage towards the M2 macrophage manifests itself by the decrease in DCFDA-ROS, NO, and H2O2 from day 5 onwards, and the flow cytometry analysis performed on day 7 for macrophage polarity further supports and validates the presence of M1 and M2 macrophage in the spheroid at day 7. These findings have implications for the development of innovative therapies that can target the inflammatory environment and cells within the TME. Tumour-induced polarization of macrophages to TAMs (Tumor-Associated Macrophages) is typically observed in advanced or late-stage cancers within the tumor microenvironment [4, 20]. This phenomenon is characterized by the activation of specific signalling pathways, such as the hypoxia-inducible factor 1-alpha (HIF-1α) pathway and hypoxia induce exosomes secretion which promote M2-like macrophage polarization which plays a crucial role in promoting tumour progression and metastasis [23, 30, 31]. In this study we observed, the presence of macrophages in the TME lead to alterations in the expression of HIF-1α, heat shock protein 70 (HSP70), and cysteine proteases cathepsin B and L which is often to correlate with poor prognosis in pathological tumour. Notably, the expression of HIF-1α was significantly increased in spheroids with macrophages, indicating a potential role for macrophages in regulating hypoxia within the TME. HSP70 and cathepsin B also showed moderate increases in expression compared to spheroids without macrophages, suggesting their involvement in cellular stress response and extracellular matrix degradation [31–33]. Additionally, cathepsin L expression was significantly elevated only in spheroids with macrophages. This finding highlights the potential role of macrophages in promoting extracellular matrix remodelling and degradation, which can facilitate tumor invasion and metastasis [22, 32–34]. Furthermore, the study evaluated the cytotoxic effects of the chemotherapeutic drug doxorubicin on CAL33 spheroids in the presence and absence of macrophages. The results showed that the presence of macrophages conferred a protective effect against the cytotoxic effects of doxorubicin, as spheroids with macrophages exhibited approximately double the IC50 value compared to spheroids without macrophages. This suggests that macrophages may suppress the efficacy of chemotherapy in cancer treatments [9, 35].

## Conclusion

Overall, the findings of this study highlight the potentially beneficial role of macrophage infiltration in spheroids for recapitulating the tumor microenvironment. They shed light on the intricate interactions between macrophages and cancer cells, suggesting their involvement in modulating key processes within the TME, such as hypoxia regulation, extracellular matrix degradation, and chemotherapeutic resistance. These findings support the potential of including macrophages in the tumor spheroids/microenvironment to improve the effectiveness of chemotherapy and develop novel therapeutic strategies targeting the TME.

### Supplementary Information

**Additional file 1:** **Figure S1.** A. Z- scan confocal microscopy (at 10X magnification) of Spheroids for CalcineAM penetration analysis. **Figure S2.** A, B. Western blot analysis of HSP70. **Figure S3.** A-B. Western blot analysis of Cathepsin B. **Figure S4.** A-B. Western blot analysis of Cathepsin L. **Figure S5.** A-B. Western blot analysis of  β-actin. **Figure S6.** A-B. Western blot analysis of HIF-1α. **Figure S7.** A, B. Western blot analysis of HIF-1α  and HSP70. **Figure S8.** A, B. Western blot analysis of Cathepsin B and Cathepsin L. **Figure S8.** A-B. Western blot analysis of β-actin.

## Data Availability

All data generated or analysed during this study are included in this published article [and its supplementary information files].

## Abbreviations

TAM: Tumor-associated macrophages
HNSCC: Head and neck squamous cell carcinoma
TME: Tumor microenvironment
ROS: Reactive oxygen species
ECM: Extracellular matrix
HSP: Heat shock protein
HIF: Hypoxia-inducible factor
OXPHOS: Oxidative phosphorylation
MEM: Minimum Essential Medium
DMEM: Dulbecco's Modified Eagle Medium
RPMI: Roswell Park Memorial Institute
FBS: Fetal bovine serum
PMA: Phorbol-12-myristate-13-acetate
DCFDA: 2',7'-Dichlorofluorescein diacetate
HRP: Horseradish peroxidase
CD: Cluster of Differentiation
CFSE: Carboxyfluoresceinsuccinimidyl ester
DAPI: 4',6-Diamidino-2-phenylindole
SISCO: SISCO Research Laboratories
AR: Analytical Reagent
ATCC: American Type Culture Collection
NCCS: National Centre for Cell Science
PBS: Phosphate-buffered saline
BSA: Bovine serum albumin
DABCO: 1,4-Diazabicyclo[2.2.2]octane
EDTA: Ethylenediaminetetraacetic acid
BCA: Bicinchoninic acid
SDS: Sodium dodecyl sulfate
PAGE: Polyacrylamide gel electrophoresis
TBS: Tris-buffered saline
ICC: Immunocytochemistry
ATP: Adenosine triphosphate
CSC: Cancer stem cells
MTS: 3-(4,5-Dimethylthiazol-2-yl)-5-(3-carboxymethoxyphenyl)-2-(4-sulfophenyl)-2H-tetrazolium
