# Supplementary material for: Macrophage infiltration in 3D cancer spheroids to recapitulate the TME and unveil interactions within cancer cells and macrophages to modulate chemotherapeutic drug efficacy
Source: BMC Cancer. 2023 Dec 7;23:1201. doi: 10.1186/s12885-023-11674-9 (PMC10701966; doi:10.1186/s12885-023-11674-9)
Supplement: Supplementary file 1 — Additional file 1: Figure S1. A. Z- scan confocal microscopy (at 10X magnification) of Spheroids for CalcineAM penetration analysis. Figure S2. A, B. Western blot analysis of HSP70. Figure S3. A-B. Western blot analysis of Cathepsin B. Figure S4. A-B. Western blot analysis of Cathepsin L. Figure S5. A-B. Western blot analysis of β-actin. Figure S6. A-B. Western blot analysis of HIF-1α. Figure S7. A, B. Western blot analysis of HIF-1α and HSP70. Figure S8. A, B. Western blot analysis of Cathepsin B and Cathepsin L. Figure S8. A-B. Western blot analysis of β-actin. [file 12885_2023_11674_MOESM1_ESM.pptx]

## Slide 1
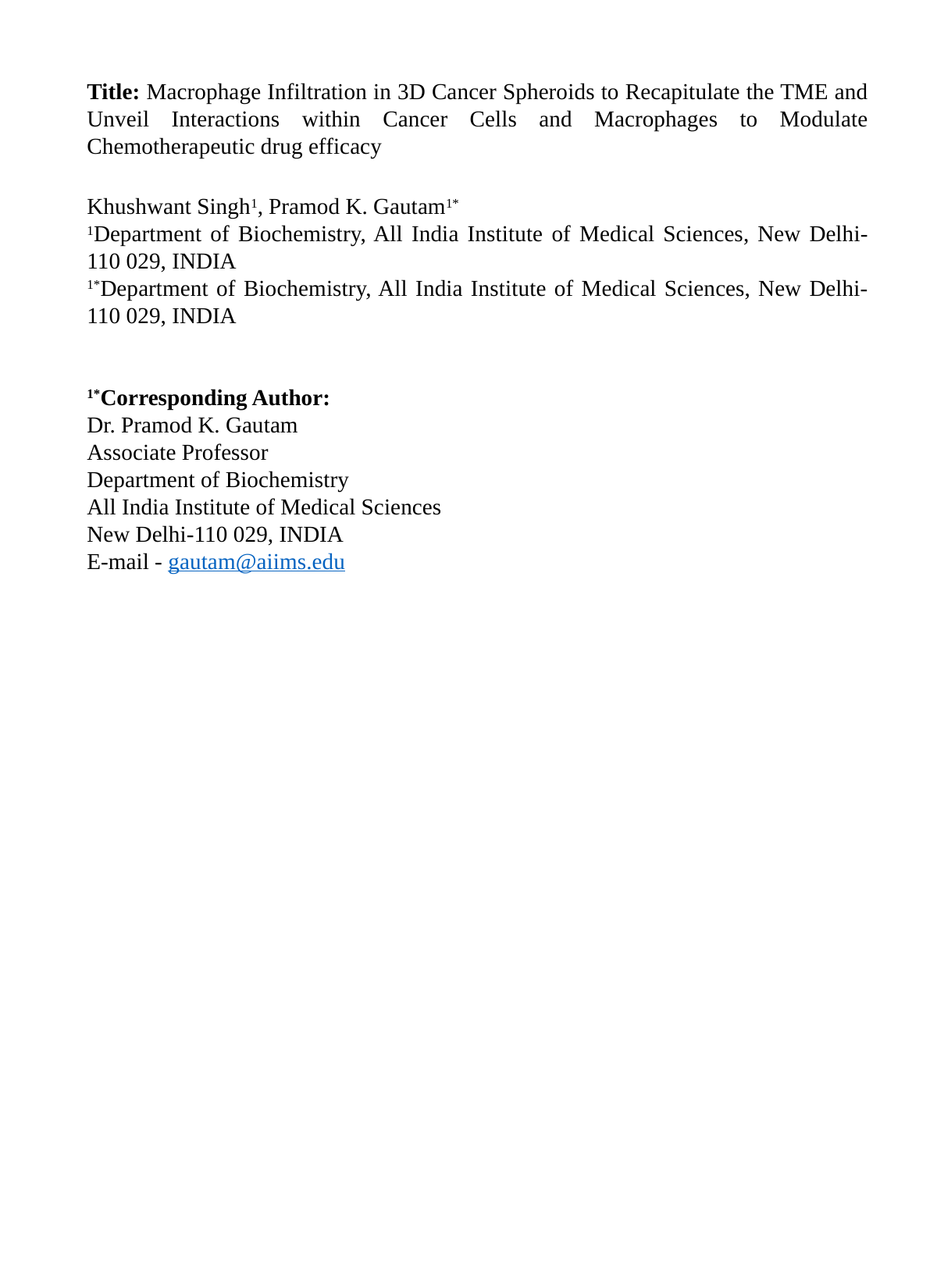

Title: Macrophage Infiltration in 3D Cancer Spheroids to Recapitulate the TME and Unveil Interactions within Cancer Cells and Macrophages to Modulate Chemotherapeutic drug efficacy
Khushwant Singh1, Pramod K. Gautam1*
1Department of Biochemistry, All India Institute of Medical Sciences, New Delhi-110 029, INDIA
1*Department of Biochemistry, All India Institute of Medical Sciences, New Delhi-110 029, INDIA
1*Corresponding Author:
Dr. Pramod K. Gautam
Associate Professor
Department of Biochemistry
All India Institute of Medical Sciences
New Delhi-110 029, INDIA
E-mail - gautam@aiims.edu

## Slide 2
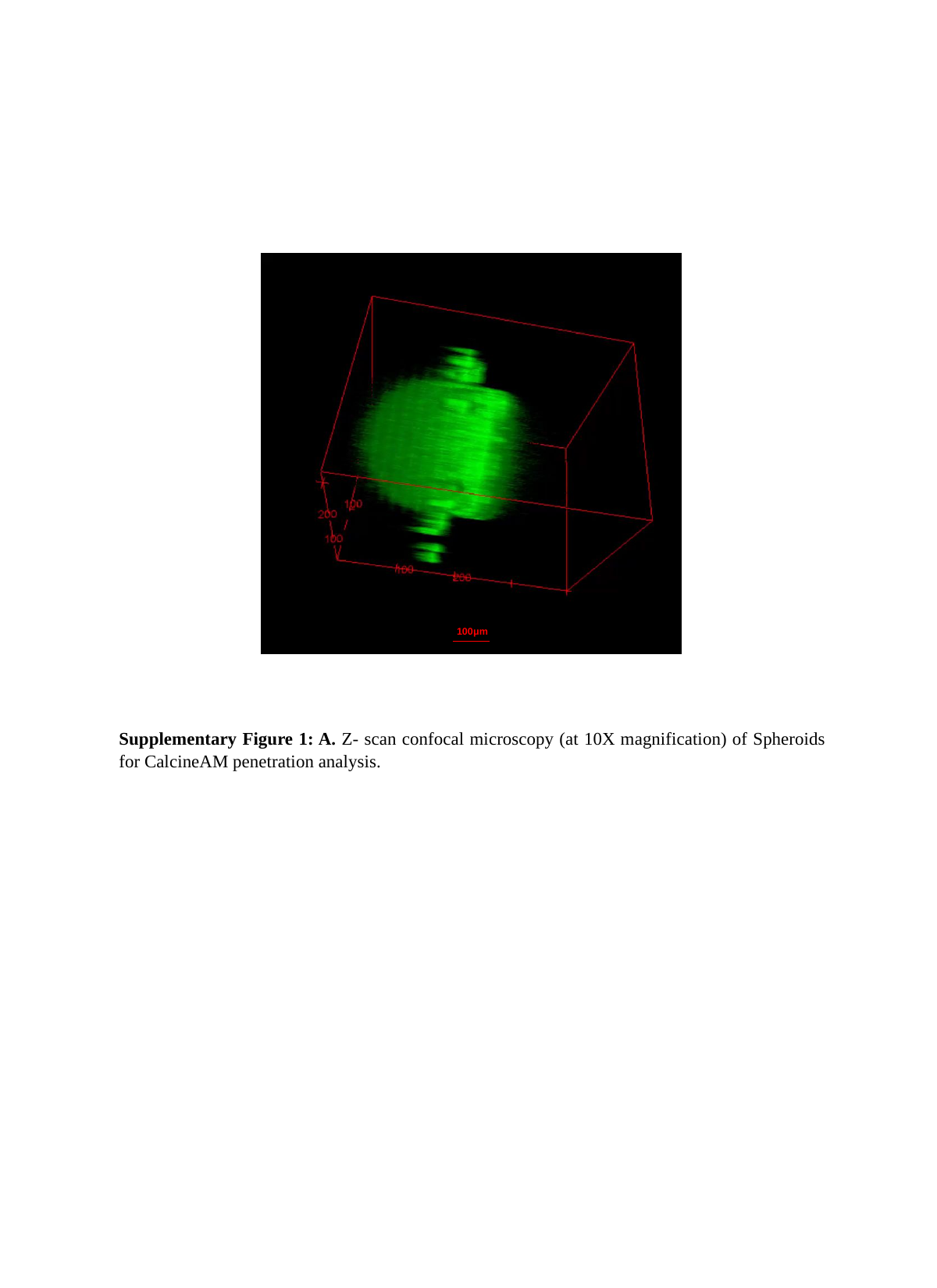

100μm
Supplementary Figure 1: A. Z- scan confocal microscopy (at 10X magnification) of Spheroids for CalcineAM penetration analysis.

## Slide 3
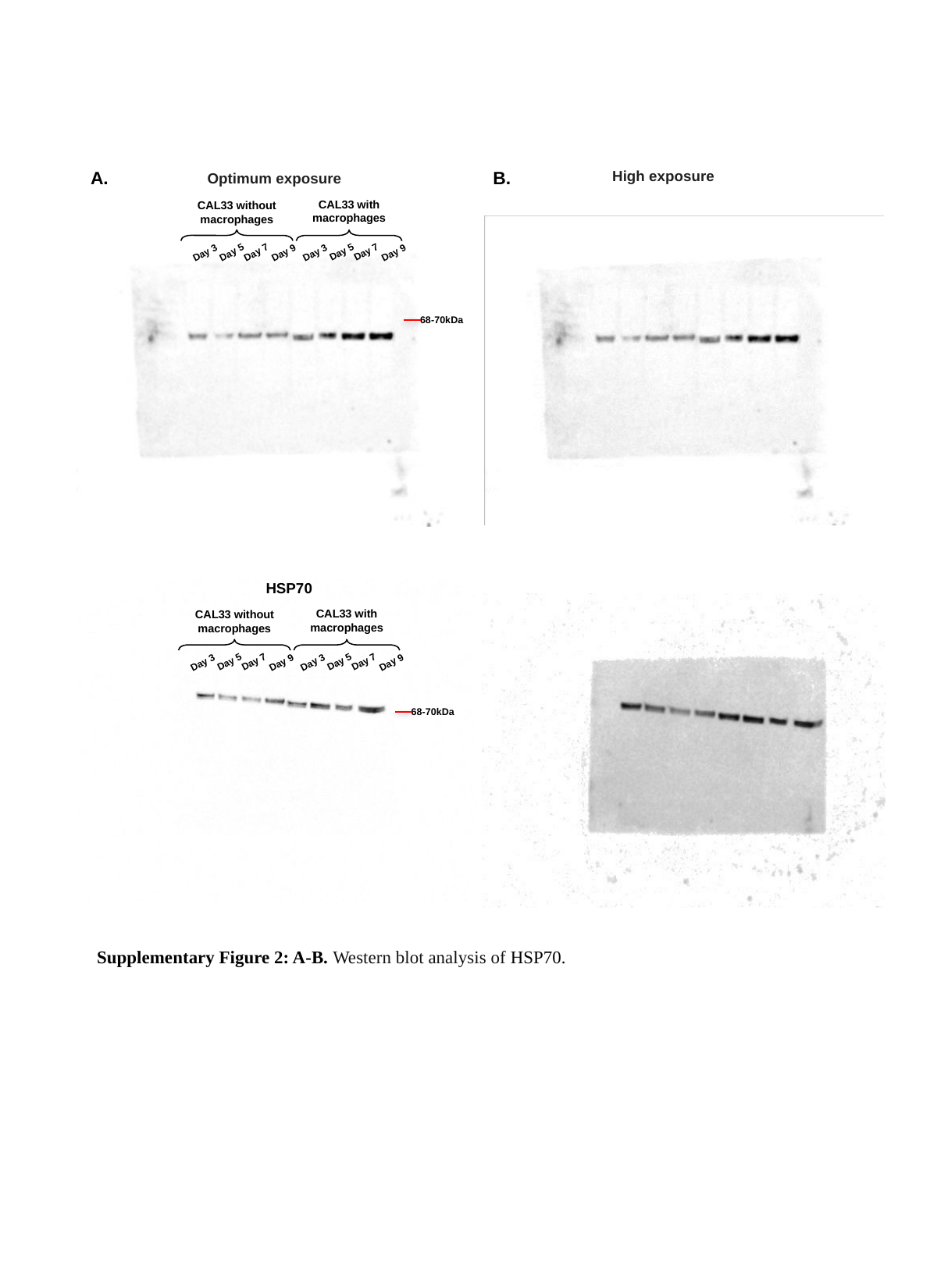

A.
B.
High exposure
Optimum exposure
CAL33 with macrophages
CAL33 without macrophages
Day 5
Day 7
Day 3
Day 9
Day 5
Day 7
Day 3
Day 9
68-70kDa
HSP70
CAL33 with macrophages
CAL33 without macrophages
Day 5
Day 7
Day 3
Day 9
Day 5
Day 7
Day 3
Day 9
68-70kDa
Supplementary Figure 2: A-B. Western blot analysis of HSP70.

## Slide 4
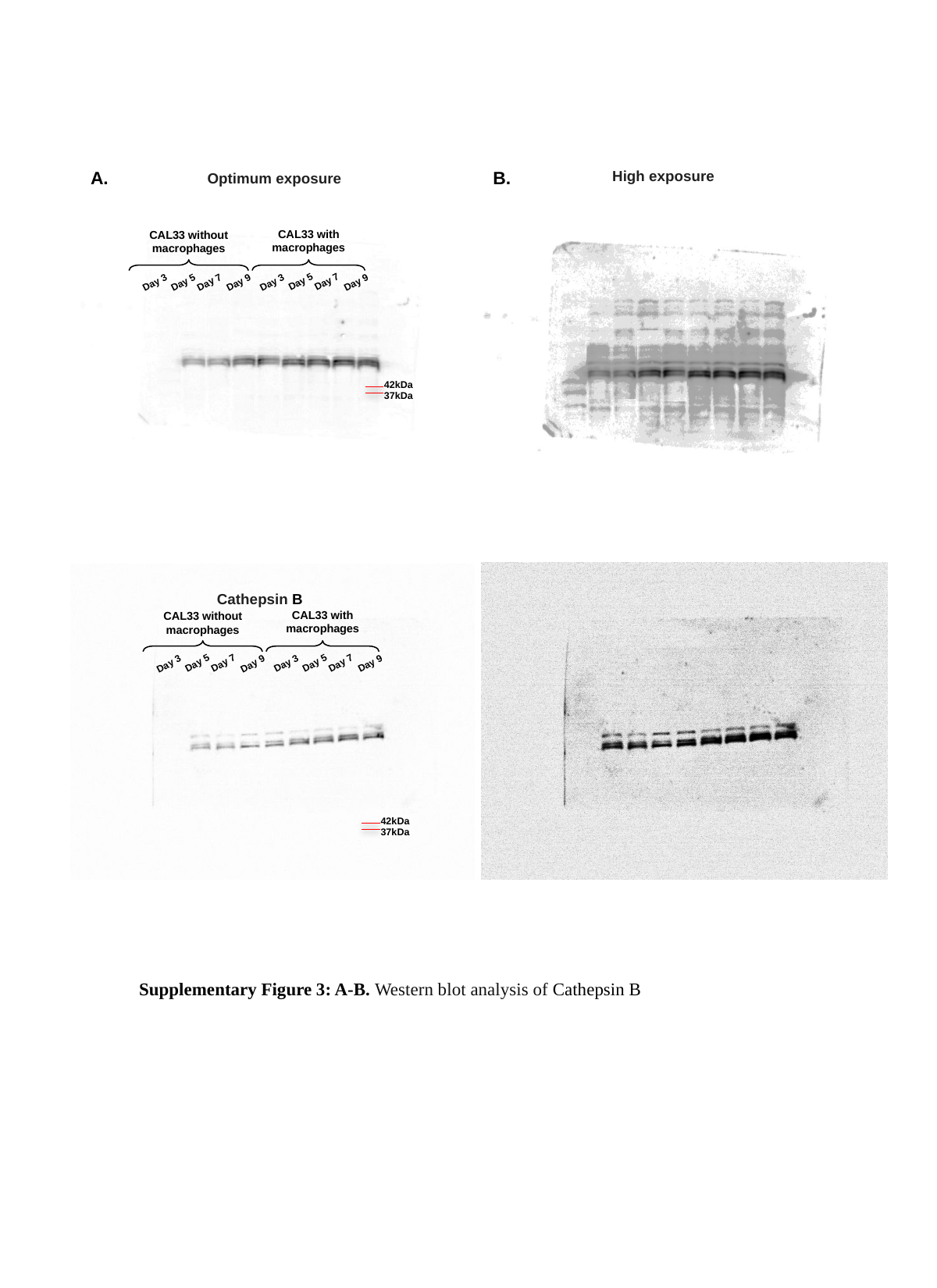

High exposure
Optimum exposure
CAL33 with macrophages
CAL33 without macrophages
Day 5
Day 7
Day 3
Day 9
Day 5
Day 7
Day 9
Day 3
42kDa
37kDa
Cathepsin B
CAL33 with macrophages
CAL33 without macrophages
Day 5
Day 7
Day 3
Day 9
Day 5
Day 7
Day 9
Day 3
42kDa
37kDa
Supplementary Figure 3: A-B. Western blot analysis of Cathepsin B
A.
B.

## Slide 5
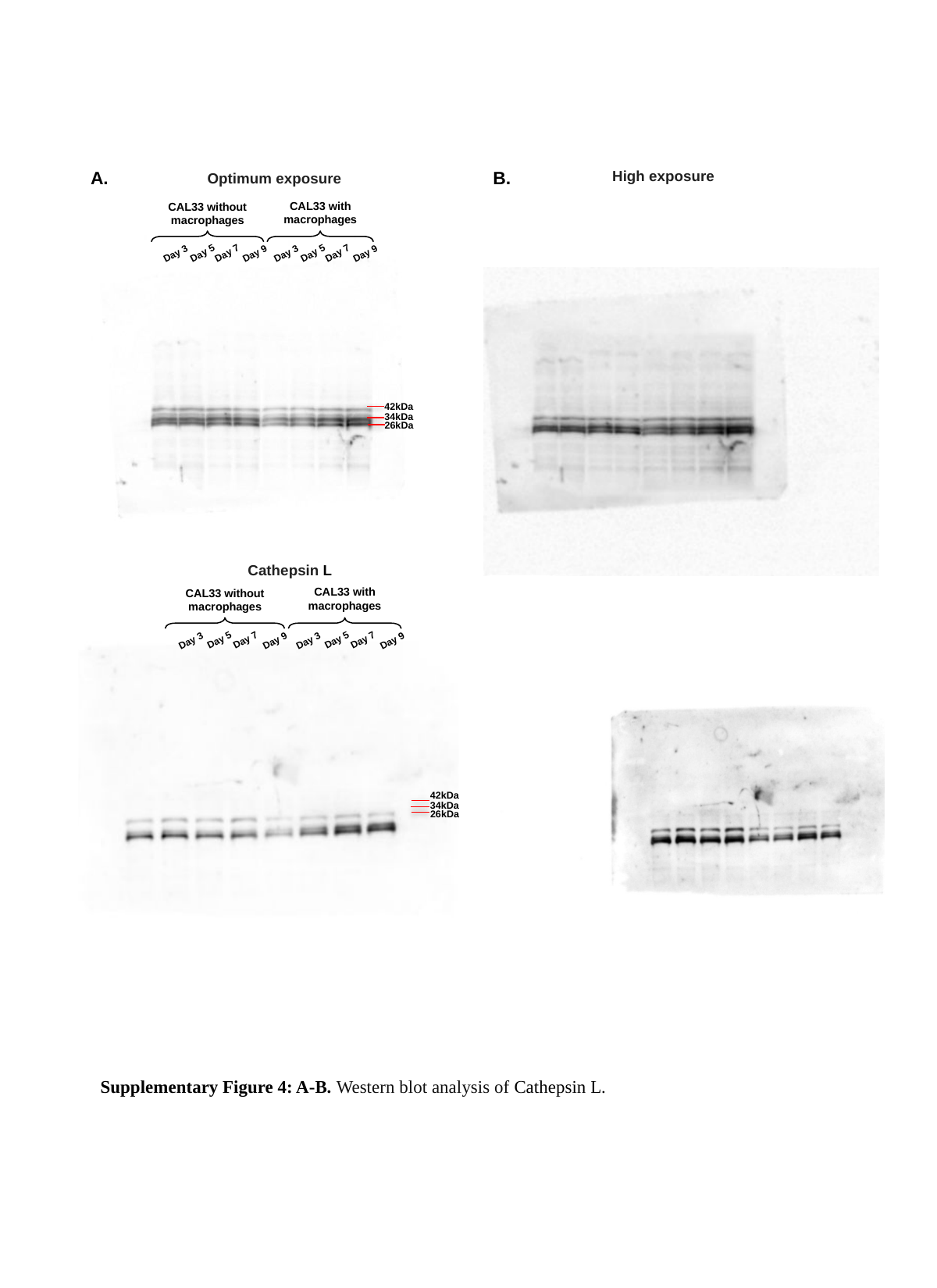

A.
B.
High exposure
Optimum exposure
CAL33 with macrophages
CAL33 without macrophages
Day 5
Day 7
Day 3
Day 9
Day 5
Day 7
Day 9
Day 3
42kDa
34kDa
26kDa
Cathepsin L
CAL33 with macrophages
CAL33 without macrophages
Day 5
Day 7
Day 3
Day 9
Day 5
Day 7
Day 9
Day 3
42kDa
34kDa
26kDa
Supplementary Figure 4: A-B. Western blot analysis of Cathepsin L.

## Slide 6
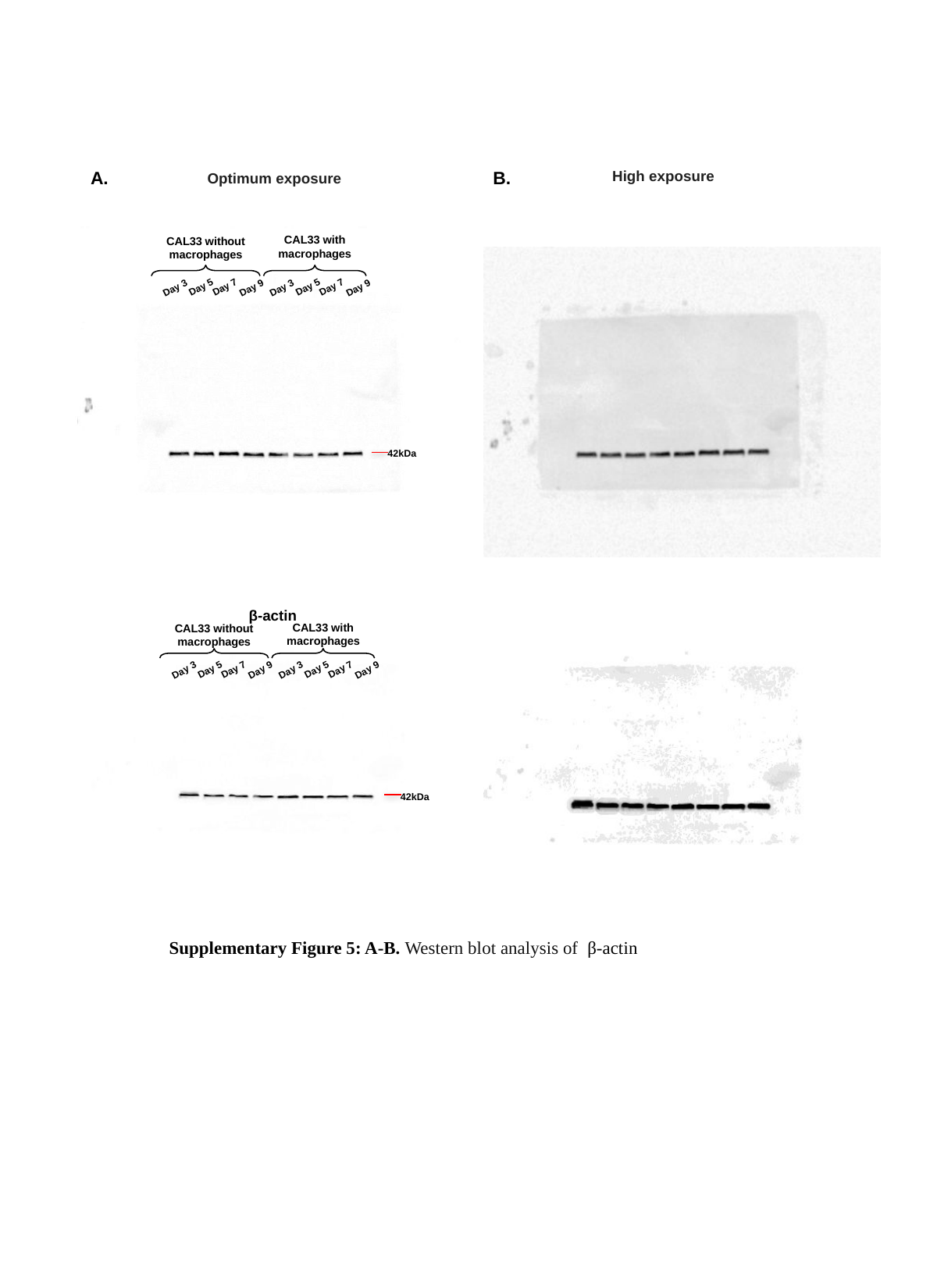

A.
B.
High exposure
Optimum exposure
CAL33 with macrophages
CAL33 without macrophages
Day 5
Day 7
Day 3
Day 9
Day 5
Day 7
Day 3
Day 9
42kDa
β-actin
CAL33 with macrophages
CAL33 without macrophages
Day 5
Day 7
Day 3
Day 9
Day 5
Day 7
Day 3
Day 9
42kDa
Supplementary Figure 5: A-B. Western blot analysis of β-actin

## Slide 7
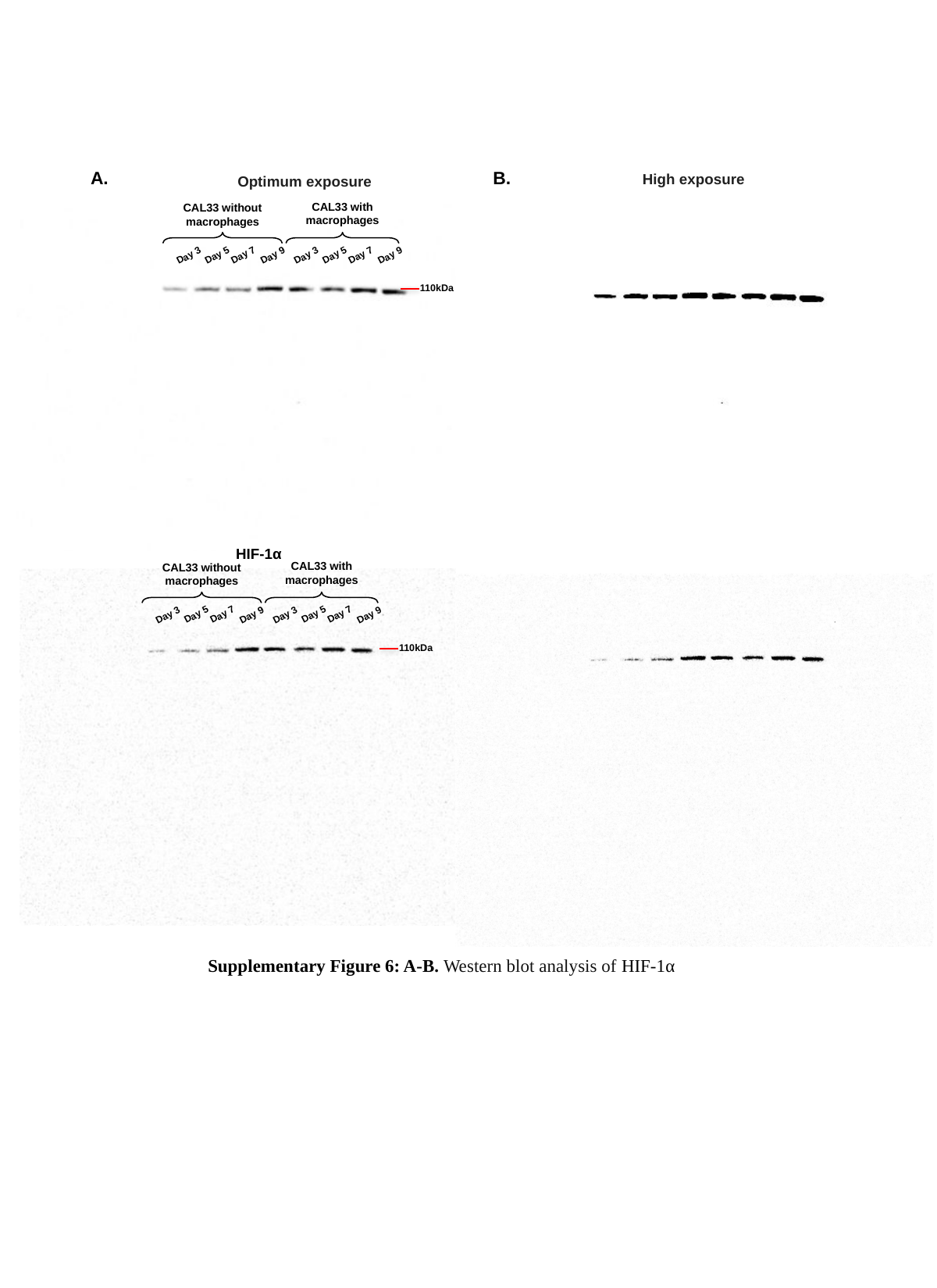

A.
B.
High exposure
Optimum exposure
CAL33 with macrophages
CAL33 without macrophages
Day 5
Day 7
Day 3
Day 9
Day 5
Day 7
Day 3
Day 9
110kDa
HIF-1α
110kDa
CAL33 with macrophages
CAL33 without macrophages
Day 5
Day 7
Day 3
Day 9
Day 5
Day 7
Day 3
Day 9
Supplementary Figure 6: A-B. Western blot analysis of HIF-1α

## Slide 8
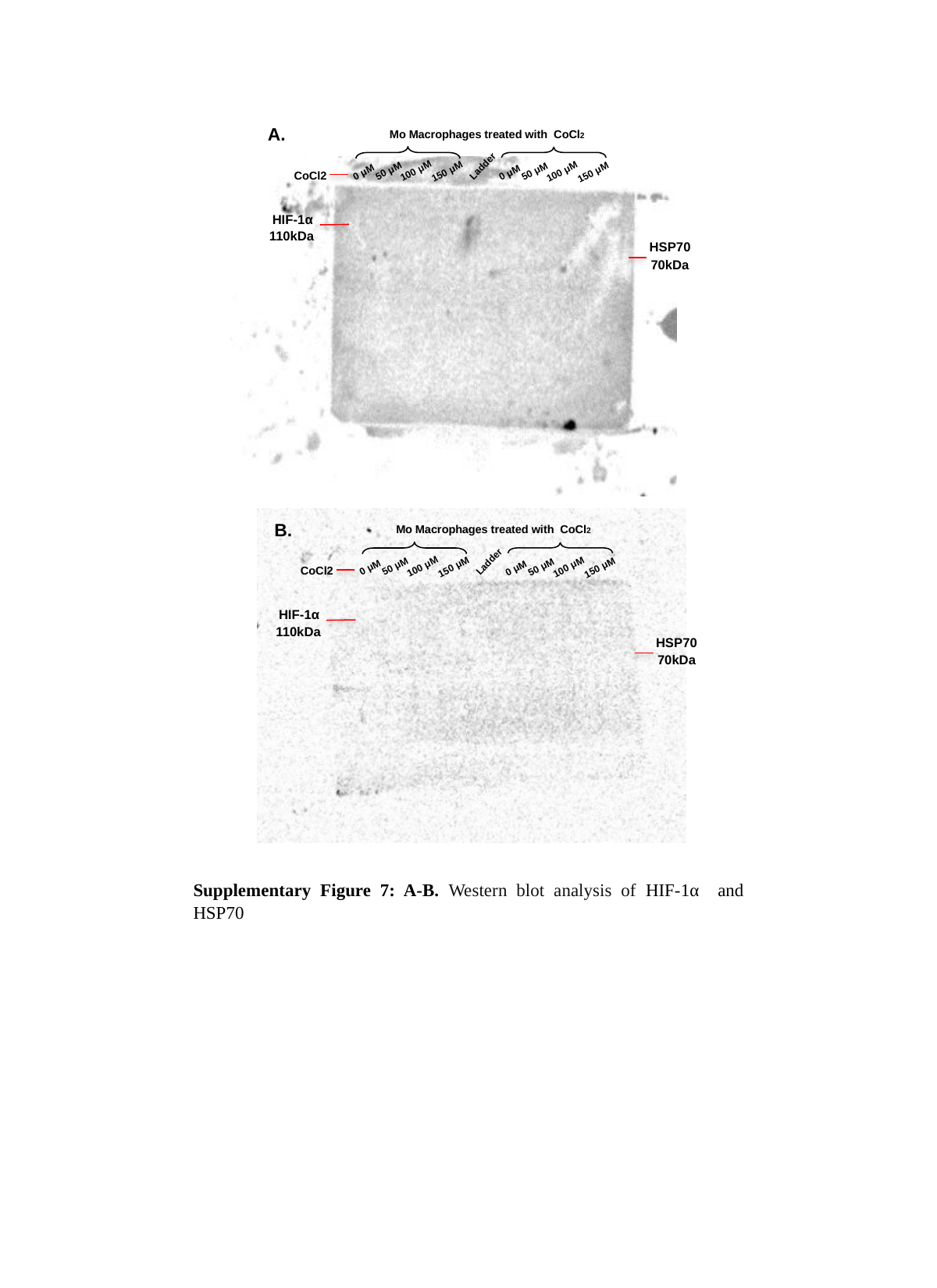

A.
Mo Macrophages treated with CoCl2
50 μM
100 μM
150 μM
0 μM
50 μM
100 μM
150 μM
0 μM
Ladder
CoCl2
HIF-1α
110kDa
HSP70
70kDa
B.
Mo Macrophages treated with CoCl2
50 μM
100 μM
150 μM
0 μM
50 μM
100 μM
150 μM
0 μM
Ladder
CoCl2
HIF-1α
110kDa
HSP70
70kDa
Supplementary Figure 7: A-B. Western blot analysis of HIF-1α and HSP70

## Slide 9
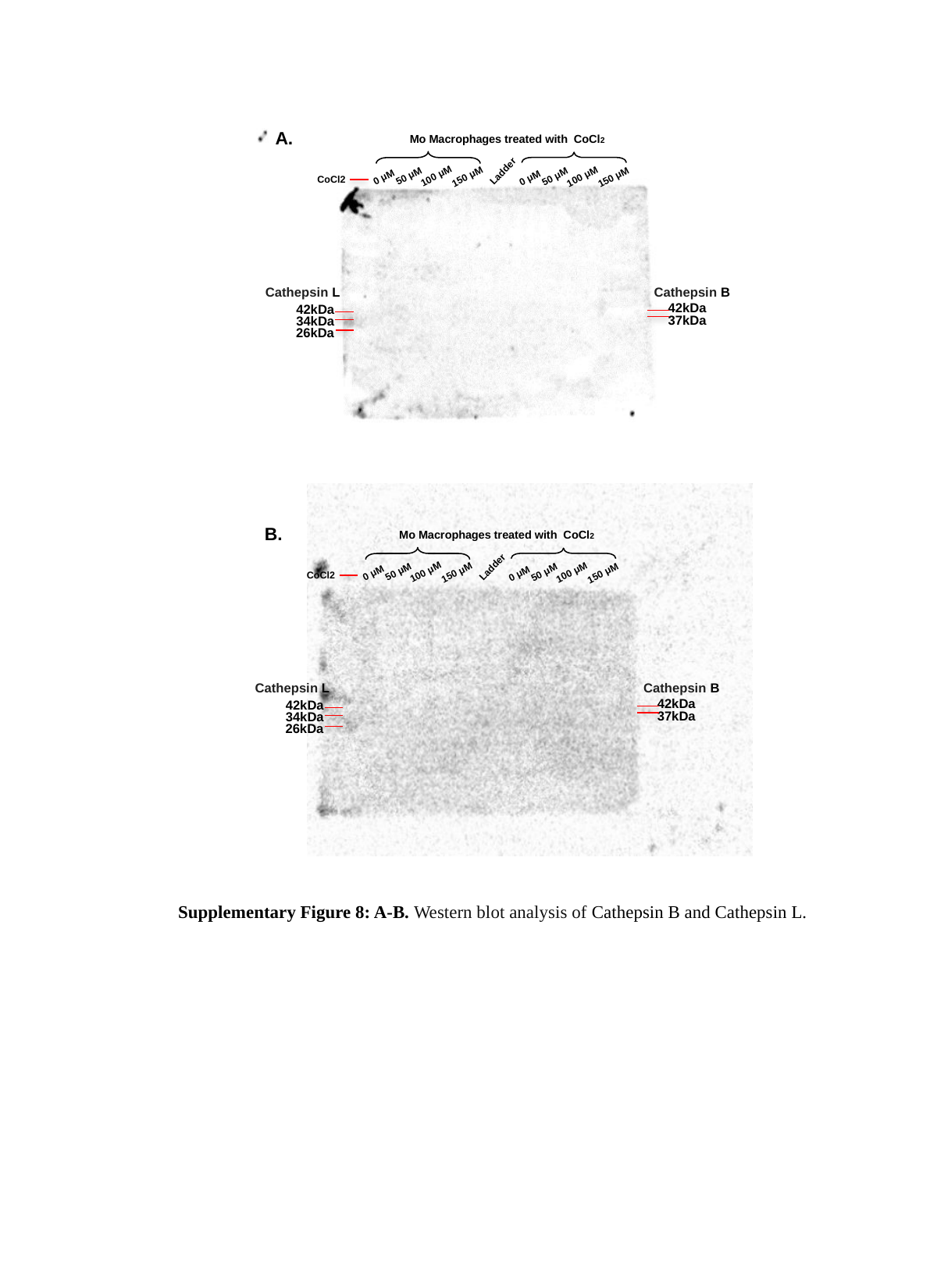

A.
Mo Macrophages treated with CoCl2
50 μM
100 μM
150 μM
0 μM
50 μM
100 μM
150 μM
0 μM
Ladder
CoCl2
Cathepsin B
42kDa
37kDa
Cathepsin L
42kDa
34kDa
26kDa
B.
Mo Macrophages treated with CoCl2
50 μM
100 μM
150 μM
0 μM
50 μM
100 μM
150 μM
0 μM
Ladder
CoCl2
Cathepsin B
42kDa
37kDa
Cathepsin L
42kDa
34kDa
26kDa
Supplementary Figure 8: A-B. Western blot analysis of Cathepsin B and Cathepsin L.

## Slide 10
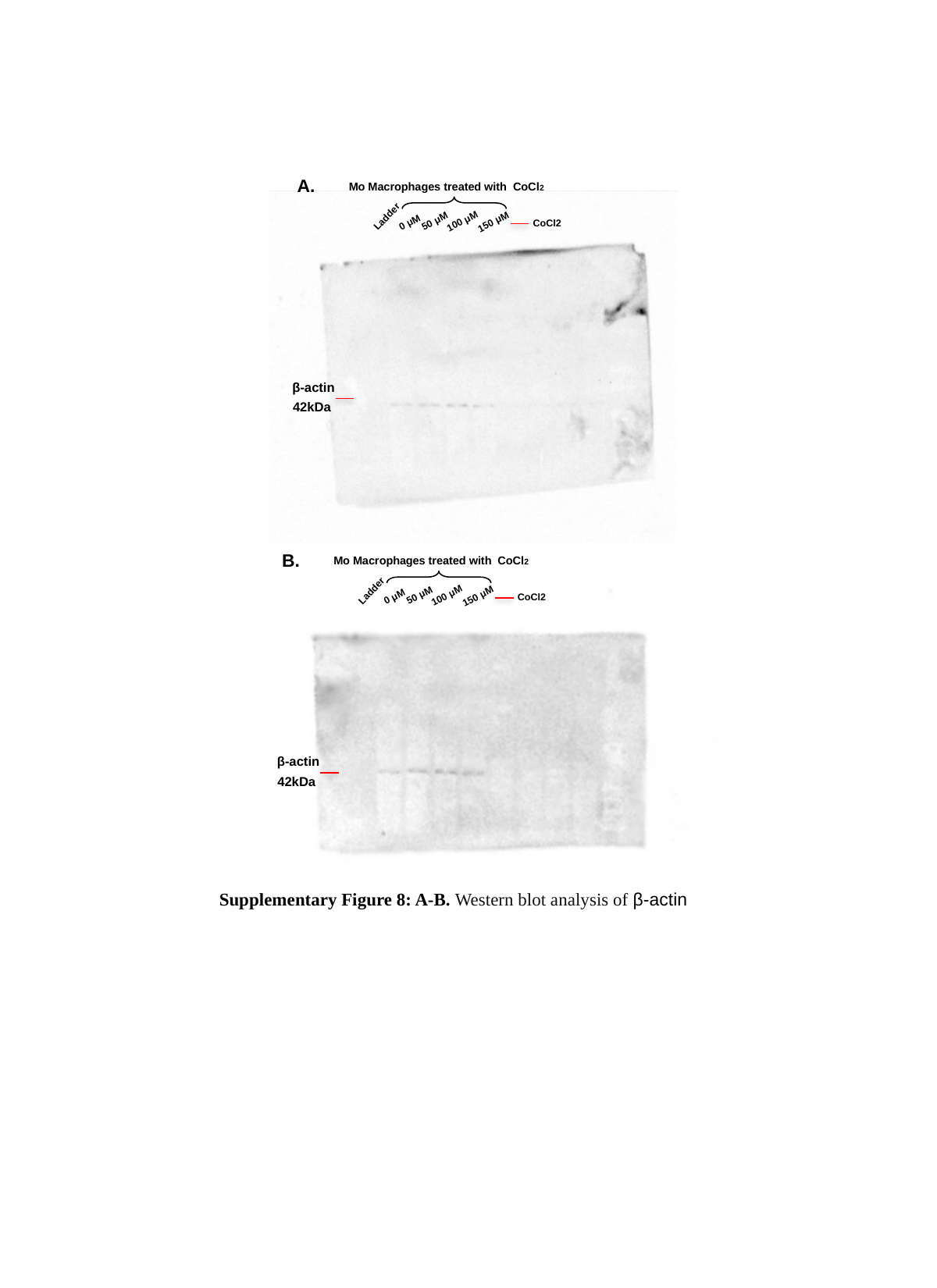

A.
Mo Macrophages treated with CoCl2
50 μM
100 μM
150 μM
0 μM
CoCl2
Ladder
β-actin
42kDa
B.
Mo Macrophages treated with CoCl2
50 μM
100 μM
150 μM
0 μM
CoCl2
Ladder
β-actin
42kDa
Supplementary Figure 8: A-B. Western blot analysis of β-actin
